# Metabolic engineering of *Escherichia coli* for the production of cinnamaldehyde

**DOI:** 10.1186/s12934-016-0415-9

**Published:** 2016-01-19

**Authors:** Hyun Bae Bang, Yoon Hyeok Lee, Sun Chang Kim, Chang Keun Sung, Ki Jun Jeong

**Affiliations:** Department of Chemical and Biomolecular Engineering (BK21 Plus Program), KAIST, 291 Daehak-ro, Yuseong-gu, Daejeon, 34141 Republic of Korea; Department of Biological Science, KAIST, 291 Daehak-ro, Yuseong-gu, Daejeon, 34141 Republic of Korea; Department of Food Science and Technology, Chungnam National University, 99 Daehak-ro, Daejeon, 17045 Republic of Korea; Institute for the BioCentury, KAIST, 291 Daehak-ro, Yuseong-gu, Daejeon, 34141 Republic of Korea

**Keywords:** *Escherichia coli*, Cinnamaldehyde, l-Phenylalanine, Metabolic engineering, Nematodes

## Abstract

**Background:**

Plant parasitic nematodes are harmful to agricultural crops and plants, and may cause severe yield losses. Cinnamaldehyde, a volatile, yellow liquid commonly used as a flavoring or food additive, is increasingly becoming a popular natural nematicide because of its high nematicidal activity and, there is a high demand for the development of a biological platform to produce cinnamaldehyde.

**Results:**

We engineered *Escherichia coli* as an eco-friendly biological platform for the production of cinnamaldehyde. In *E. coli*, cinnamaldehyde can be synthesized from intracellular l-phenylalanine, which requires the activities of three enzymes: phenylalanine-ammonia lyase (PAL), 4-coumarate:CoA ligase (4CL), and cinnamoyl-CoA reductase (CCR). For the efficient production of cinnamaldehyde in *E. coli*, we first examined the activities of enzymes from different sources and a gene expression system for the selected enzymes was constructed. Next, the metabolic pathway for l-phenylalanine biosynthesis was engineered to increase the intracellular pool of l-phenylalanine, which is a main precursor of cinnamaldehyde. Finally, we tried to produce cinnamaldehyde with the engineered *E. coli*. According to this result, cinnamaldehyde production as high as 75 mg/L could be achieved, which was about 35-fold higher compared with that in the parental *E. coli* W3110 harboring a plasmid for cinnamaldehyde biosynthesis. We also confirmed that cinnamaldehyde produced by our engineered *E. coli* had a nematicidal activity similar to the activity of commercial cinnamaldehyde by nematicidal assays against *Bursaphelenchus xylophilus*.

**Conclusion:**

As a potential natural pesticide, cinnamaldehyde was successfully produced in *E. coli* by construction of the biosynthesis pathway and, its production titer was also significantly increased by engineering the metabolic pathway of l-phenylalanine.

**Electronic supplementary material:**

The online version of this article (doi:10.1186/s12934-016-0415-9) contains supplementary material, which is available to authorized users.

## Background

Nematodes are small organisms that belong to the phylum Nematoda and can be subdivided into five categories: fungivorous (phagocytize fungi), bacterivorous (phagocytize bacteria), corrosive (disassemble organics), predatory (prey on small nematodes), and plant-parasitic nematodes (damage plants). Among them, plant-parasitic nematodes occur naturally in many agricultural soils of the world, causing severe economic damage [1, 2]. Root-knot nematodes, which are classified as plant-parasitic nematodes, are the most deleterious to agricultural products, leading to annual crop yield losses of 10–27 % [3]. In particular, crops like cucumber, watermelon, tomato, carrot, ginseng, and lettuce can be seriously damaged by this nematode. Because root-knot nematodes only gradually harm the underground roots of plants, it is difficult to assess the damage until the entire plant is pulled out or the crop dies [4, 5].

To prevent agricultural crop and plant damage, various pest control systems such as the use of resistant cultivars, and physical or chemical controls, have been employed, but the effectiveness of these methods is limited because of restrictions by climate conditions [6, 7]. Therefore, chemical pesticides are used worldwide to control nematodes effectively. Nematicides are chemical pesticides that eliminate parasitic nematodes on plants. They can be employed with success, although currently there are several disadvantages: (i) it is necessary to pay particular attention to the chemical pesticide used in many susceptible plants and crops, (ii) non-selectivity of the pesticide may occasionally cause elimination of other organisms in addition to nematodes, and (iii) if the pathogen remains after treatment, the damage may be severely exacerbated [8, 9]. To substitute for current agricultural chemicals, natural pesticides, which are derived from plants and microbes, have been researched and developed intensively. Biological pesticides are often less toxic and safer to plants than chemicals, and they do not require extensive reclamation. To date, various molecules derived from nature have been demonstrated which have nematicidal activity [10, 11]. Among them, the specific molecule cinnamaldehyde, which is present in cinnamon bark oil, is attractive as a pesticide because of its potent nematicidal activity [12, 13]. Besides being the main flavoring agent of cinnamon, cinnamaldehyde can be used in a number of different applications in addition to its use as a nematicide; it can be used for treating hepatitis B, diabetes, dementia, and also it has anti-cancer and anti-fungal activities [14, 15]. Cinnamaldehyde is currently obtained by chemical synthesis from benzaldehyde and acetaldehyde or by direct extraction from cinnamon bark oil. However, there are disadvantages with these methods: (i) it is difficult to separate other cinnamaldehyde derivatives and stereoisomers during its chemical production, and (ii) extraction requires the logging and consumption of plants [16]. Therefore, there is an urgent need for the development of an eco-friendly and efficient biological system to produce cinnamaldehyde.

In this study, we sought to engineer *Escherichia coli* as a microbial cell factory for the production of cinnamaldehyde by construction of the biosynthesis pathway for the production of cinnamaldehyde. Based on in vitro assays of enzyme activities, three biosynthetic enzymes, phenylalanine-ammonia lyase (PAL), 4-coumarate:CoA ligase (4CL), and cinnamoyl-CoA reductase (CCR) were cloned into gene expression constructs. We also manipulated metabolic pathways in *E. coli* to increase the intracellular pool of l-phenylalanine, which is the main precursor of cinnamaldehyde. Using our engineered *E. coli* strain with cinnamaldehyde biosynthesis system, we examined cinnamaldehyde production and compared it to that of the parental *E. coli* strain harboring same cinnamaldehyde biosynthesis system. The nematicidal activity of cinnamaldehyde produced by our engineered *E. coli* strain was also determined against the nematode, *Bursaphelenchus xylophilus,* which causes pine wilt.

## Results and discussion

### Selection of enzymes for cinnamaldehyde biosynthesis

**Fig. 1:**
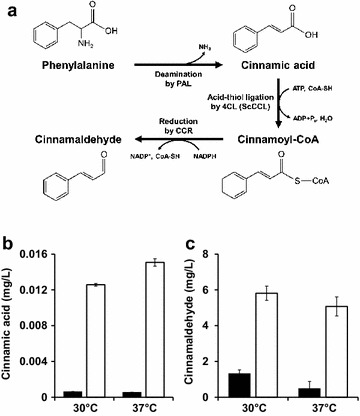
Biosynthesis of cinnamaldehyde and in vitro assay of synthesis enzymes. **a** Three enzymatic reactions (PAL, 4CL, and CCL) for the biosynthesis of cinnamaldehyde from l-phenylalanine. **b** In vitro assay of PAL from *A. thaliana* (AtPAL1, *black*) and *S. maritimus* (SmPAL, *white*) at 30 and 37 °C. **c** In vitro assay of 4CL and CCL at 30 and 37 °C. Two combinations including (i) 4CL from *A. thaliana* (At4CL1) and CCR from *A. thaliana* (AtCCR), and (ii) CCL from *S. coelicolor* (ScCCL) and CCR from *A. thaliana* (AtCCR) were mixed with cinnamic acid and cinnamaldehyde production was analyzed. *Error bars* represent standard deviation of the mean (n = 2)

Cinnamaldehyde can be synthesized from l-phenylalanine and its biosynthesis requires three enzymatic reactions: (i) deamination of l-phenylalanine into cinnamic acid by phenylalanine-ammonia lyase (PAL, EC 4.3.1.24), (ii) acid-thiol ligation of cinnamic acid to cinnamoyl-CoA by 4-coumarate:CoA ligase (4CL, EC 6.2.1.12), and (iii) reduction of cinnamoyl-CoA to cinnamaldehyde by cinnamoyl-CoA reductase (CCR, EC 1.2.1.44) (Fig. 1a) [17–20]. PAL is a ubiquitous enzyme that can be found in many plants, fungi, and some bacteria, and it harbors diverse activities and specificities according to the origin of the enzyme [18, 21]. We examined two PAL enzymes, one from the plant *Arabidopsis thaliana* (AtPAL) and another from the bacterium *Streptomyces maritimus* (SmPAL), for their suitability to produce cinnamic acid in *E. coli*. In case of AtPAL, there are four isomers including AtPAL1 to AtPAL4, and it was previously reported that most of them (AtPAL1, 2, and 4) have similarly higher activities than that of AtPAL3 to l-phenylalanine as a substrate [22], so we selected AtPAL1 as a representative from plant source. Their kinetic constants (K_m_) were reported to be 68 and 23 μM, respectively [22, 23]. Each His-tagged PAL enzyme was produced in *E. coli* BL21(DE3) and purified following procedures as described in “Methods”. Both enzymes, AtPAL1 (78 kDa) and SmPAL (56 kDa), were successfully purified (Additional file 1: Figure S1). Although the expression level of AtPAL1 was not so high which band could not be seen in the lanes 1 and 2 of SDS–PAGE, the band of AtPAL1 could be clearly seen after affinity column chromatography in the lane 3 in which the concentrated elute was loaded. The equivalent molarity of each purified PAL enzyme was incubated with the same amount of l-phenylalanine (as a substrate), and the production titer of cinnamic acid was quantified by high-performance liquid chromatography (HPLC) (Additional file 2: Figure S2). As shown in Fig. 1b, SmPAL exhibited significantly higher activity (21-fold at 30 °C reaction and 27-fold at 37 °C reaction) than those of AtPAL1.

We also examined two different 4CL enzymes, one from *Streptomyces coelicolor* (ScCCL) and another from *A. thaliana* (At4CL), for their suitability to convert cinnamic acid to cinnamaldehyde in *E. coli*. In case of At4CL, it is known that there are 14 putative isoforms (At4CL1–At4CL14) [24, 25]. Among 14 isoforms, At4CL1–3 have similar activities to cinnamate while rest of isoforms do not [19]. Therefore, we selected At4CL1 as a representative. Also, we used CCR enzyme from *A. thaliana* (AtCCR1) for conversion of cinnamoyl-CoA into cinnamaldehyde [26]. Each 4CL enzyme (At4CL1 and ScCCL [27]) was tested in combination with CCR enzyme from *A. thaliana* (AtCCR). All enzymes were successfully purified with high purity (Additional file 1: Figure S1). To compare the enzymatic activities of At4CL1 and ScCCL, two reactions, (i) At4CL1 with AtCCR and (ii) ScCCL with AtCCR, were prepared and mixed with cinnamic acid as a substrate. After incubating at 30 and 37 °C, cinnamaldehyde titer was analyzed by HPLC. As shown in Fig. 1c, the combination of ScCCL and AtCCR resulted in a higher production titer of cinnamaldehyde (4.4-fold at 30 °C reaction and 10.4-fold at 37 °C reaction) than the combination of At4CL1 and AtCCR. Based on these results, we constructed cinnamaldehyde biosynthesis system in *E. coli* as described below using the following enzymes: SmPAL, ScCCL, and AtCCR.

### Construction of cinnamaldehyde biosynthesis pathway in *E. coli*

**Fig. 2:**
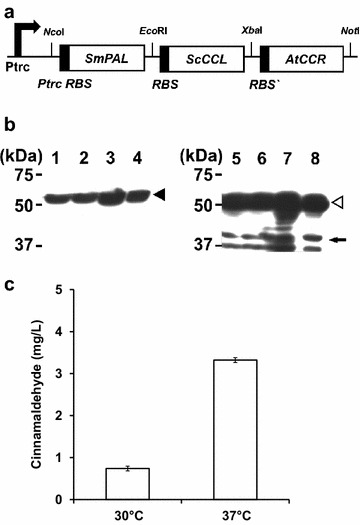
The construction of expression system, production of each enzymes and cinnamaldehyde in *E. coli*. **a** The schematic diagram of plasmid pHB-CAD for the expression of three synthesis genes (SmPAL, ScCCL, and AtCCR genes) under the IPTG-inducible *trc* promoter (P_trc_). RBS means the ribosome binding site for translation and restriction enzyme sites were designated. **b** Western blot analysis of gene expression under two different temperature (30 and 37 °C). For the detection of SmPAL (*lanes* *1*–*4*), anti-FLAG-HRP antibody was used and for the detection of ScCCL and AtCCR (*lanes* *5*–*8*), anti-His-HRP antibody was used. *Lanes 1*, *3*, *5*, and *7* indicate total protein fraction, and *lanes 2*, *4*, *6*, and *8* indicates the soluble protein fractions. *Lanes 1*, *2*, *5*, and *6* indicate samples at 30 °C, and *lanes 3*, *4*, *7*, and *8* indicate samples at 37 °C. Symbols: *Closed arrowhead*, SmPAL; *open arrowhead*, ScCCL; *solid arrow*, AtCCR. **c** HPLC analysis of cinnamaldehyde produced under two different temperatures. *Error bars* represent standard deviation of the mean (n = 3)

To produce cinnamaldehyde in *E. coli*, SmPAL, ScCCL, and AtCCR genes were cloned into pTrc99A in the following order: SmPAL, ScCCL, and AtCCR (yielding pHB-CAD) (Fig. 2a). *E. coli* W3110 harboring pHB-CAD was cultivated at two different temperatures (30 and 37 °C) to find the optimal temperature for cinnamaldehyde production. The expression of enzymes was analyzed by SDS–PAGE, followed by western blot analysis as described in “Methods”. At both temperatures, all enzymes were expressed well and highly soluble (Fig. 2b). Although each enzyme was expressed at a different level, the expression of each enzyme was marginally better at 37 °C than at 30 °C. Also, analysis of cinnamaldehyde titer produced in the culture medium using HPLC revealed that cinnamaldehyde production titer was 4.5-fold higher at 37 °C than at 30 °C (Fig. 2c). We supposed that the higher production titer of cinnamaldehyde at 37 °C was caused by increased expression level of all biosynthetic enzymes at 37 °C actively, even though these enzymes are active at 30 °C [23, 27]. Hereafter, all cultivations for the production of cinnamaldehyde were performed at 37 °C.

### Strain engineering to increase the intracellular pool of l-phenylalanine

**Fig. 3:**
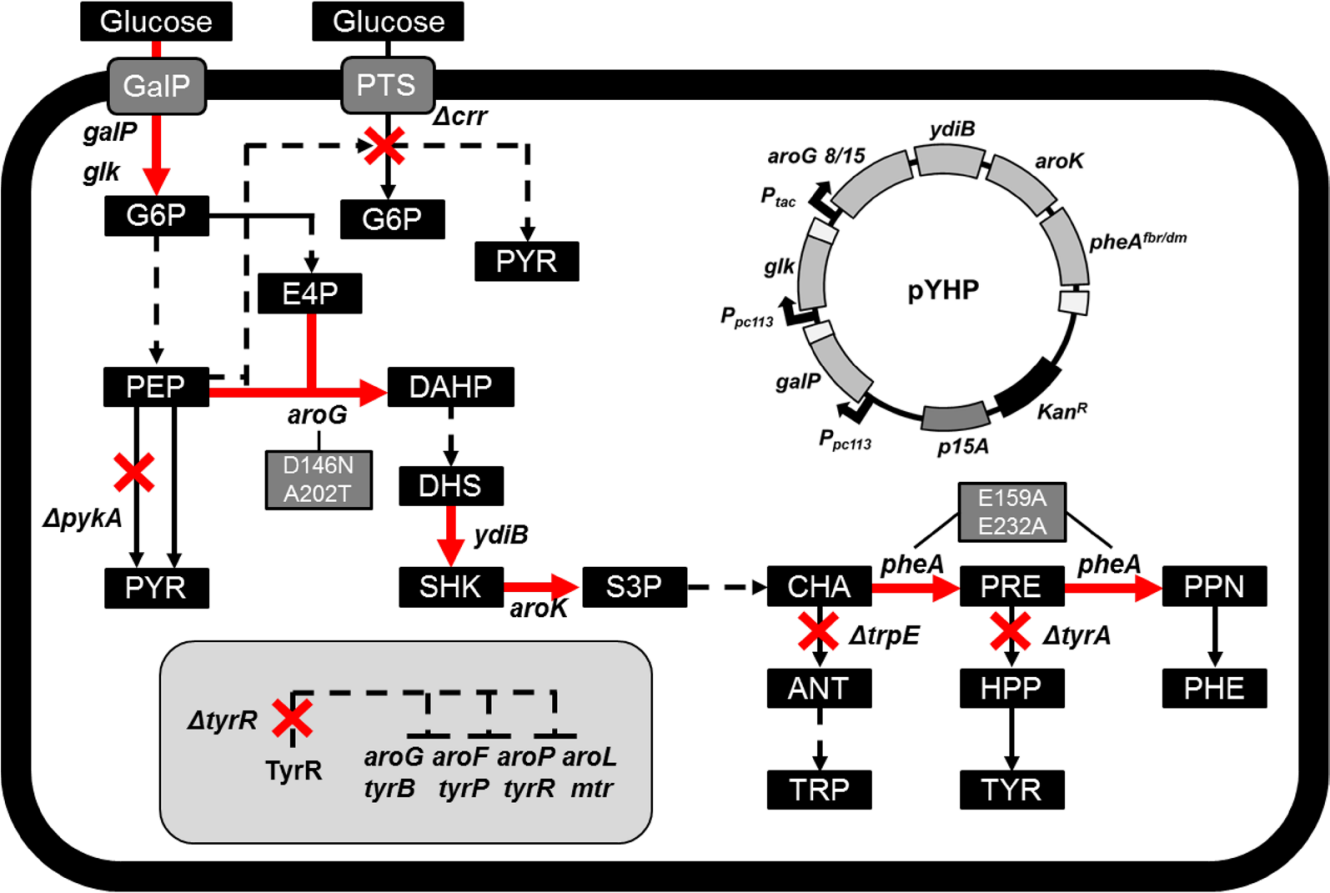
The schematic diagram for strain engineering to increase the pool of l-phenylalanine in *E. coli* W3110. The symbol “*X*” indicates the corresponding gene deletion. The *red-color arrows* indicate overexpression of the relevant genes (*galP, glk, aroG, ydiB, aroK, pheA*) via plasmid (pYHP)-based expression system

For the biosynthesis of cinnamaldehyde, l-phenylalanine is required as an essential precursor [17]. Therefore, to enhance cinnamaldehyde production, it is desirable to increase the intracellular pool of l-phenylalanine. For this purpose, we rationally re-designed *E. coli* to produce l-phenylalanine more. Using available metabolic and regulatory information as a guide, we engineered *E. coli* W3110 as follows: (i) deletion of the *crr* gene that encodes EIIA^Glc^ protein related to glucose-specific phosphoenolpyruvate phosphotransferase system (PTS) for moderating the substrate uptake rate, decreasing metabolite overflow, and precursor enrichment, (ii) deletion of the *tyrR* gene to alleviate the tight regulation of the TyrR regulon which contains aromatic amino acid (AAA) synthesis genes, (iii) deletion of the *trpE* (anthranilate synthase component) and *tyrA* [chorismate (CHA) mutase/prephenate dehydrogenase] genes to prevent the loss of carbon flow into competing pathways (biosynthesis of l-tryptophan and l-tyrosine), and (iv) deletion of the *pykA* (pyruvate kinase A) gene to enrich the precursor and balance the flux between growth and l-phenylalanine production (Fig. 3). Based on the above scheme, five sequential knockout mutants of *E. coli* W3110 were developed (YHP01–YHP05) (Table 1).

**Table 1:** Bacterial strains and plasmids used in this study

	Description	Reference or source
Strain		
XL1-Blue	*recA1 endA1 gyrA96 thi-1 hsdR17 supE44 relA1 lac* [*F*′ *proAB lacI* ^*q*^ *Z ΔM15 Tn10* (*Tet* ^*r*^)]	Stratagene^1^
BL21(DE3)	*E. coli B F* ^−^ *dcm ompT hsdS*(*r*_*B*_^−^ *m*_*B*_^−^) *gal λ*(*DE3*)	Novagen^2^
W3110	*F* ^−^ *λ* ^−^ *rph-1 INV*(*rrnD, rrnE*)	CGSC no. 4474^3^
YHP01	W3110 *Δcrr*	This study
YHP02	W3110 *Δcrr ΔtyrR*	This study
YHP03	W3110 *Δcrr ΔtyrR ΔtrpE*	This study
YHP04	W3110 *Δcrr ΔtyrR ΔtrpE ΔtyrA*	This study
YHP05	W3110 *Δcrr ΔtyrR ΔtrpE ΔtyrA ΔpykA*	This study
Plasmid		
pTac15k	Km^R^, p15A origin, *tac* promoter, 4.0 kb	[54]
pTrc99A	Amp^R^, pBR322 origin, *trc* promoter, 4.2 kb	Pharmacia^4^
pUC19	Amp^R^, pMB1 origin, *lac* promoter, 2.6 kb	New England biolabs^5^
pET-22b	Amp^R^, pBR322 origin, *T7* promoter, 5.5 kb	EMD chemicals^6^
pHB-I01	pET-22b derivatives, P_T7_–*At*PAL1 (N-term 8× His-tag)	This study
pHB-I02	pET-22b derivatives, P_T7_–*At*4CL1 (N-term 8× His-tag)	This study
pHB-I03	pET-22b derivatives, P_T7_–*At*CCR (N-term 8× His-tag)	This study
pHB-I04	pET-22b derivatives, P_T7_–*Sm*PAL (N-term 8× His-tag)	This study
pHB-I05	pET-22b derivatives, P_T7_–*Sc*CCL (N-term 8× His-tag)	This study
pHB-CA	pTrc99A derivatives, P_trc_–*Sm*PAL (C-term FLAG-tag)	This study
pHB-CAD	pTrc99A derivatives, P_trc_–*Sm*PAL (C-term FLAG-tag)–ScCCL (N-term 8× His-tag)–AtCCR (N-term 8× His-tag)	This study
pUC19G	pUC19, *aroG8/15*	This study
pUC19B	pUC19, *ydiB*	This study
pUC19K	pUC19, *aroK*	This study
pUC19A	pUC19, *pheA* ^*fbr, dm*^	This study
pTac15kG	pTac15k, P_tac_–*aroG8/15*	This study
pTac15kGB	pTac15k, P_tac_–*aroG8/15*–*ydiB*	This study
pTac15kGBK	pTac15k, P_tac_–*aroG8/15*–*ydiB*–*aroK*	This study
pTac15kGBKA	pTac15k, P_tac_–*aroG8/15*–*ydiB*–*aroK*–*pheA* ^*fbr, dm*^	This study
pYH	pTac15k, P_tac_–*aroG8/15*–*ydiB*–*aroK*–*pheA* ^*fbr, dm*^, *Sac*II region modification	This study
pPpc113	pUC19, synthetic BBa_J23113 Anderson promoter (Ppc113)	This study
pTrc99A-mod	pTrc99A, MCS modification (*Spe*I, *Not*I)	Lab stock
pGlk	pTrc99A-mod derivatives, Ppc113–*glk*–T_lpp_	This study
pGalP	pTrc99A-mod derivatives, Ppc113–*galP*–T_lpp_	This study
pYH-glk	pYH derivatives, Ppc113–*glk*–T_lpp_	This study
pYHP	pYH-glk derivatives, Ppc113–*galP*–T_lpp_	This study
pCW611	Amp^R^, λ-red recombinase expression plasmid under *ara*-inducible BAD promoter, cre-loxP recombinase system under *lacUV5* promoter, temp sensitive origin	[50]
pEcmuloxC	Amp^R^, Cm^R^, pUG6 derivatives, lox66-cat-lox71	[55]

^1^Stratagene cloning systems, La Jolla, CA, USA

^2^Novagen, inc., Madison, WI, USA

^3^The coli genetic stock center, Yale University, USA

^4^Pharmacia biotech, Uppsala, Sweden

^5^New England biolabs, inc., Beverly, MA, USA

^6^EMD chemicals, Gibbstown, NJ, USA

First, a glucose-specific phosphoenolpyruvate PTS was inactivated by deletion of the *crr* gene in *E. coli* W3110 strain, yielding *E. coli* YHP01. Although this disrupts the main system for glucose internalization, YHP01 can still grow in defined media containing glucose as a sole carbon source because the mannose-specific PTS and galactose permease can continue to internalize glucose into the cytoplasm [28, 29]. Bypassing the PTS results in an increased pool of phosphoenolpyruvate (PEP), which ultimately facilitates l-phenylalanine synthesis [29]. Next, we deleted the *tyrR* gene in YHP01 to yield YHP02 strain. TyrR protein is a regulator of the TyrR regulon, which contains eight genes involved in the biosynthesis of AAA [30]. Its deletion can also increase the l-phenylalanine pool by alleviating the tight regulation of genes related with AAA synthesis. We next sequentially deleted *trpE* and *tyrA* genes starting with YHP02 strain, yielding YHP03 and YHP04 strains, respectively. In the biosynthesis pathway of AAAs, a final branch point occurs wherein chorismate can be converted to l-phenylalanine, l-tryptophan or l-tyrosine by PheA, TrpE or TyrA enzymes, respectively [31, 32]. The deletions of *trpE* and *tyrA* genes can prevent the loss of carbon into competing pathways for l-tryptophan and l-tyrosine biosynthesis. Finally, we deleted the *pykA* gene in YHP04 to yield YHP05 strain. The *pykA* gene encodes pyruvate kinase A (PykA), which constitutes the second PEP-consuming step. By deleting the *pykA* gene, more PEP can be used in the shikimate pathway and consequently, more amount of l-phenylalanine can be produced [33]. In each strain (YHP01–YHP05), deletion of genes was verified by PCR and agarose gel electrophoresis (Additional file 3: Figure S3).

**Fig. 4:**
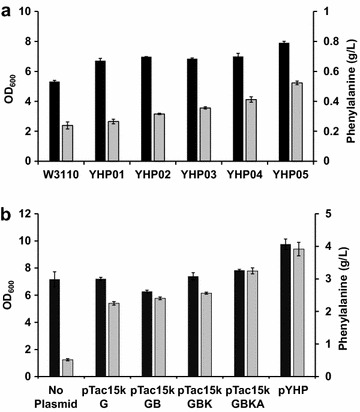
Comparison of final optical density (*black*) and l-phenylalanine production (*gray*) in 48 h flask cultivation. **a** All five engineered *E. coli* strains (YHP01 to YHP05) and the parental *E. coli* W3110 were cultivated and cell growth (OD_600_) and l-phenylalanine production titers were compared. **b** *E. coli* YHP05 strain harboring different plasmids (pTac15kG series or pYHP) were cultivated and cell growth (OD_600_) and l-phenylalanine production titers were compared. *Error bars* represent standard deviation of the mean (n = 3)

All engineered *E. coli* strains, including YHP01–YHP05 and the parental *E. coli* W3110, were cultivated in shaking flasks containing semi-defined media, and cell growth and l-phenylalanine production were compared. After cultivation for 48 h, all engineered strains grew slightly better than W3110 strain; in particular, *E. coli* YHP05 strain exhibited the highest cell density (Fig. 4a). A previous study also observed that inactivation of the PTS and Pyk isozymes increased carbon flux to biomass formation because of the effect of conserving reduced amounts of intermediate metabolites resulting from decreased glucose uptake and catabolism [33]. We also analyzed the production titer of l-phenylalanine in the culture supernatant by HPLC. In the parental W3110 strain, the production titer of l-phenylalanine was 0.24 g/L, but the titer of l-phenylalanine was gradually increased in the engineered *E. coli* strains (Fig. 4a). *E. coli* YHP05, in which *crr*, *tyrR*, *trpE*, *tyrA*, and *pykA* genes were deleted, exhibited the highest l-phenylalanine production titer (0.52 g/L), which was 2.2-fold higher than that of parental *E. coli* W3110. Therefore, we decided to use *E. coli* YHP05 strain for further engineering.

### Plasmid-based overexpression system to increase the intracellular pool of l-phenylalanine

Starting with *E. coli* YHP05 strain, production of l-phenylalanine was further improved by plasmid-based gene overexpression. The feedback inhibition in the l-phenylalanine synthesis pathway by shikimate and l-phenylalanine was reduced by overexpressing isozymes or introducing mutations in enzymes involved in the shikimate pathway as follows: (i) overexpression of the 3-deoxy-d-arabinoheptulosonate 7-phosphate (DAHP) synthase gene which is encoded in *aroG* with engineering (AroG8/15), (ii) overexpression of the *ydiB* and *aroK* genes which encode shikimate dehydrogenase and shikimate kinase for enhancing the carbon flow into the shikimate pathway, (iii) overexpression of the *pheA* gene encoding CHA mutase/prephenate dehydratase with engineering for improvement of substrate binding affinity (PheA^fbr, dm^), and (iv) overexpression of the *galP* (galactose permease) and *glk* (glucokinase) genes to facilitate glucose uptake (Additional file 4: Figure S4).

First, to alleviate feedback inhibition, the plasmid pTac15kG was constructed, which contained the engineered *aroG8/15* gene. AroG is the major enzyme involved in the synthesis of DAHP, but AroG is completely inhibited by l-phenylalanine (as low as 0.1 mM) [32, 34]. It is known that the introduction of two mutations (D146N and A202T) resulted in resistance to feedback inhibition without affecting its high specific activity (AroG8/15) [35], so we overexpressed this mutant AroG8/15 enzyme. Next, two plasmids (pTac15kGB and pTac15kGBK) were constructed to overexpress *ydiB* and *aroK* genes along with *aroG8/15* gene, respectively. Metabolic flux to the shikimate pathway can be enhanced when shikimate dehydrogenase (YdiB) and shikimate kinase (AroK) are overexpressed [36, 37]. Also, pTac15kGBKA plasmid was subsequently constructed to overexpress *pheA* gene which encodes chorismate mutase/prephenate dehydratase with mutations. In this construct, we amplified only the first 300 amino acids of wild-type PheA (PheA^fbr^), which excludes the regulatory domain; therefore, PheA^fbr^ is weakly influenced by feedback inhibition. In addition, because PheA^fbr^ has a higher K_m_ value than wild-type PheA, reflecting its decreased binding affinity to the substrate [38], we introduced two mutations (E159A and E232A) in PheA^fbr^ to enhance its substrate-binding affinity, yielding PheA^fbr, dm^ [39]. Lastly, pYHP plasmid was constructed to overexpress *galP* and *glk* genes additionally, which encode galactose permease and glucokinase, respectively. Both enzymes facilitate glucose uptake [40].

After the construction of five plasmids, including pTac15kG, pTac15kGB, pTac15kGBK, pTac15kGBKA, and pYHP (Table 1), each plasmid was transformed into *E. coli* YHP05. After cultivation for 48 h in shaking flasks containing semi-defined media, cell growth and the production titer of l-phenylalanine were analyzed. All cells grew well, and particularly *E. coli* YHP05 harboring pYHP grew to a marginally higher cell density (OD_600_ = 9.76) than the others (Fig. 4b). We also analyzed the production titer of l-phenylalanine in the culture supernatant by HPLC. Overexpression of the *aroG8/15* gene (pTac15kG) resulted in a significant increase in l-phenylalanine production (2.25 g/L) compared with YHP05 harboring no plasmid (0.52 g/L) (Fig. 4b). Subsequent overexpression of other genes resulted in a serial increase in the production titer of l-phenylalanine and, *E. coli* YHP05 harboring pYHP exhibited the highest l-phenylalanine production titer (3.91 g/L) (Fig. 4b). The effect of pYHP plasmid resulted in a significant increase in l-phenylalanine production as high as 16.4-fold [comparison between W3110 (no plasmid) and YHP05 (pYHP)] and 7.5-fold [comparison between YHP05 (no plasmid) and YHP05 (pYHP)], respectively. In the cultivation of *E. coli* YHP05 harboring pYHP, l-phenylalanine yield on glucose and productivity were 0.270 g/g and 0.082 g/L/h, respectively (Additional file 5: Figure S5). Thus, in the engineered *E. coli* YHP05 harboring pYHP, the pool of l-phenylalanine was significantly improved. Liu et al. previously reported the production of l-phenylalanine in *E. coli* as high as 47 g/L [41]. However, that record could be achieved in the fed-batch cultivation (15 L scale) and they employed the co-expression of l-phenylalanine transporter (YddG) for the efficient production of l-phenylalanine into culture medium. In our work, we did not introduce the YddG because the ultimate goal of our work was not a production of l-phenylalanine but production of cinnamaldehyde. Although the titer of l-phenylalanine achieved in our work was not the high record, we thought it was enough high for the production of cinnamaldehyde. Therefore, we decided to use this engineered strain for the production of cinnamaldehyde.

### Cinnamaldehyde production in the engineered *E. coli*

**Fig. 5:**
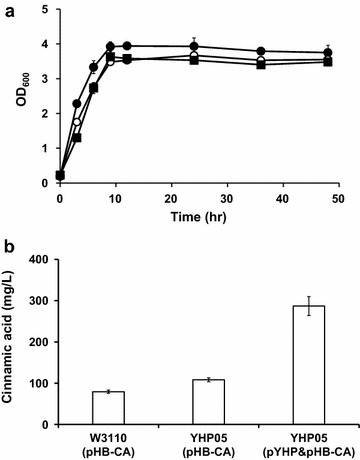
Cell growth and cinnamic acid production in flask cultivation. **a** Time profiles of cell growths (OD_600_). Symbols: *Closed circle*, *E. coli* W3110 (pHB-CA); *open circle*, *E. coli* YHP05 (pHB-CA); *closed square*, *E. coli* YHP05 (pHB-CA and pYHP). **b** Production titer of cinnamic acid in each strain. *Error bars* represent standard deviation of the mean (n = 3)

Using the engineered *E. coli* YHP05 harboring pYHP, we first examined the production of cinnamic acid. For this experiment, the plasmid pHB-CA, which contains *Sm*PAL gene under IPTG-inducible *trc* promoter was constructed (Table 1). *E. coli* YHP05 harboring both pHB-CA and pYHP was cultivated in flasks for 48 h and the production titer of cinnamic acid was analyzed. *E. coli* YHP05 and *E. coli* W3110 harboring pHB-CA (without pYHP) were also examined as controls. Growth patterns of all cells were similar (Fig. 5a) and, *E. coli* W3110 and YHP05 harboring pHB-CA produced 79 and 108 mg/L of cinnamic acid, respectively (Fig. 5b). *E. coli* YHP05 harboring both pHB-CA and pYHP exhibited significantly improved production titer (287 mg/L), which was 3.6-fold and 2.7-fold higher than those of *E. coli* W3110 and YHP05 harboring pHB-CA, respectively (Fig. 5b). To the best of our knowledge, this production titer was also 1.5-fold higher than the highest level (186 mg/L) reported in *E. coli* [42, 43]. This result clearly indicates that the elevating the amount of l-phenylalanine in the engineered *E. coli* strain positively contributes to increase cinnamic acid production.

**Fig. 6:**
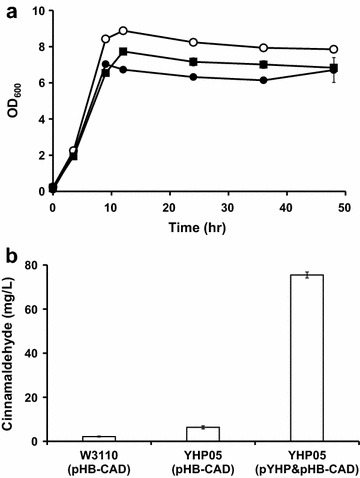
Cell growth and cinnamaldehyde production in flask cultivation. **a** Time profiles of cell growths (OD_600_). Symbols: *Closed circle*, *E. coli* W3110 (pHB-CAD); *open circle*, *E. coli* YHP05 (pHB-CAD); *closed square*, *E. coli* YHP05 (pHB-CAD and pYHP). **b** Production titer of cinnamaldehyde in each strain. *Error bars* represent standard deviation of the mean (n = 3)

As a main goal, we examined cinnamaldehyde production in the engineered *E. coli* YHP05 strain, which was transformed with pHB-CAD and pYHP. Also, *E. coli* YHP05 and *E. coli* W3110 harboring pHB-CAD (without pYHP) were examined as controls. The growth of cell was similar (Fig. 6a), and three cinnamaldehyde biosynthetic enzymes were well expressed in all examined cells (Additional file 6: Figure S6). After flask cultivation for 48 h, culture supernatants were collected and the production titers of cinnamaldehyde were determined by HPLC. *E. coli* W3110 (pHB-CAD) and *E. coli* YHP05 (pHB-CAD) exhibited cinnamaldehyde production titer as high as 2.18 and 6.3 mg/L, respectively (Fig. 6b). On the contrary, *E. coli* YHP05 (pHB-CAD and pYHP) exhibited significantly higher production titer (75 mg/L), which was 35-fold higher than that of *E. coli* W3110 (pHB-CAD).

### Nematicidal activity of cinnamaldehyde produced by engineered *E. coli*

**Fig. 7:**
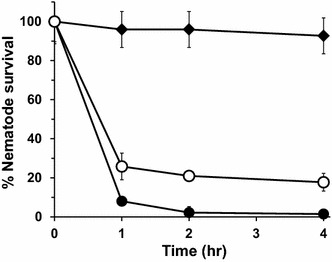
Graph of nematode percentage alive (%) after treatment of cinnamaldehyde. Symbols: *Closed diamond*, culture supernatant of *E. coli* W3110 as a negative control; *closed circle*, 60 mg/L commercial and purified cinnamaldehyde as positive control; *open circle*, 60 mg/L culture supernatant with cinnamaldehyde produced in *E. coli* YHP05 harboring pYHP and pHB-CAD. *Error bars* represent standard deviation of the mean (n = 2)

To evaluate the nematicidal activity of cinnamaldehyde produced in culture medium, nematodes, *B. xylophilus,* were treated with cinnamaldehyde following procedures as described in “Methods” [12]. The culture supernatant of *E. coli* YHP05 harboring pYHP and pHB-CAD was diluted until the final concentration of cinnamaldehyde was 60 mg/L, and nematodes were treated with the diluted culture supernatant. After 1 and 4 h, only 26 % and below 18 % of nematodes survived, respectively (Fig. 7). As a positive control, nematodes were treated with commercially available and purified cinnamaldehyde at an equivalent concentration (60 mg/L). After 4 h, nearly all nematodes (95 %) were killed. These results indicated that the nematicidal activities were similar between commercially purchased cinnamaldehyde and produced one in this study. As a negative control, the culture supernatant of *E. coli* W3110 was also tested and as expected, nearly all nematodes survived (>92 %) after 4 h.

## Conclusions

As a potential natural pesticide, the demand for cinnamaldehyde is increasing, and thus the development of an eco-friendly biological system for the industrial production of cinnamaldehyde to replace traditional chemical synthesis processes has become increasingly important [16]. In this study, we constructed the cinnamaldehyde biosynthesis pathway in *E. coli* strain, which can potentially be used as an eco-friendly production platform. Although each enzyme (PAL, 4CL, and CCR) in the cinnamaldehyde biosynthesis pathway has been well-studied in the aspects of their enzymatic properties [19, 22–27], there is still no report about the combination of all three enzymes for biological production of cinnamaldehyde from glucose. To the best of our knowledge, this is the first report that cinnamaldehyde was biologically synthesized in *E. coli*. By introducing enzymes for cinnamaldehyde biosynthesis and metabolically engineering of *E. coli* for the increased production of the main precursor l-phenylalanine, engineered strain *E. coli* YHP05 harboring pHB-CAD and pYHP produced cinnamaldehyde at titer as high as 75 mg/L, which represented a 35-fold improvement in titer compared to that of the wild-type strain. Through extensive engineering of metabolic pathways to increase the intracellular l-phenylalanine pool, we achieved the production of l-phenylalanine at titer as high as 3.91 g/L with a high glucose yield of 0.270 g/g [41, 44]. In addition, through further optimization of culture conditions on an industrial scale, we suggest that our engineered strain may be useful for the industrial-scale production of l-phenylalanine. Our general strategy for engineering *E. coli* can be adopted to engineer strains for the production of other amino acids (particularly AAAs). Although we succeeded to produce cinnamaldehyde using *E. coli* strains in flask cultivations, the production titer (75 mg/L) was not sufficient to warrant their commercialization, which require improvements to achieve gram-scale titer. Therefore, fed-batch cultivation conditions in a lab-scale have to be selected and the study of culture optimization including media compositions and feeding strategies, etc. will be our next work [45, 46]. In addition, the low conversion rate of cinnamaldehyde from l-phenylalanine may be attributed to the relatively poor activities of the biosynthetic enzymes: SmPAL, ScCCL, or AtCCR. Further engineering of the activities of each enzyme or the development of protein scaffolds for the modular control of pathway flux [47, 48] may aid in improving enzymatic reactions and achieving much higher production titer.

## Methods

### Bacterial strains and plasmids

Detailed information about *E. coli* strains and plasmids used in this study are described in Table 1. *E. coli* XL1-Blue was used for gene cloning and plasmid maintenance. *E. coli* BL21(DE3) was used for protein expression and purification. *E. coli* W3110 strain was used as the primary host for the production of cinnamaldehyde. The pTrc99A vector was used for the expression of cinnamaldehyde biosynthetic genes, and the pTac15k vector was used for the expression of l-phenylalanine biosynthetic genes. All DNA plasmid manipulations, including restriction enzyme digestions, ligations, and transformations, were performed following standard protocols [49]. All restriction enzymes were purchased from Enzynomics™ (Daejeon, Republic of Korea). Polymerase chain reactions (PCR) were performed using a C1000™ Thermal Cycler (Bio-Rad, Richmond, CA, USA) with PrimeStar HS polymerase (Takara Bio, Shiga, Japan). Detailed procedures for the construction of plasmids are described in Additional file 7, Additional file 8: Table S1, and Additional file 9: Table S2.

### Strain construction

In *E. coli* W3110 strain, *crr*, *tyrR*, *trpE*, *tyrA*, and *pykA* genes were deleted using a rapid one-step inactivation method [50]. The integrated knockout system vector (pCW611) was used to disrupt the desired genes in the chromosomal DNA of *E. coli* W3110, and the chloramphenicol-resistance gene (Cm^R^) was used for selection. Chromosomal gene deletions were performed sequentially, and the genotypes of deletion mutants (YHP01 to YHP05) are described in Table 1. Detailed procedures for gene knockout are provided in Additional file 10 and Additional file 8: Table S1 .

### Cultivation condition

*E. coli* cells were grown in Luria–Bertani (LB) liquid medium (10 g/L tryptone, 5 g/L yeast extract, and 10 g/L NaCl) or on LB agar plates (LB containing 1.5 % (w/v) agar). When necessary, the following concentrations of antibiotics were added to the medium to maintain plasmids: 100 μg/mL ampicillin (Amp) and 40 μg/mL kanamycin (Kan). Flask cultivation for the production of l-phenylalanine was performed using modified fermentation medium previously [51–53]. The composition of the fermentation medium is: 20 g/L glucose, 3 g/L KH_2_PO_4_, 5 g/L (NH_4_)_2_SO_4_, 1 g/L NaCl, 1.5 g/L sodium citrate, 0.015 g/L CaCl_2_∙2H_2_O, 3 g/L MgSO_4_∙7H_2_O, 0.01125 g/L FeSO_4_∙7H_2_O, 0.075 g/L Thiamine-HCl, 0.3 g/L l-tyrosine (L-Tyr), 0.03 g/L l-tryptophan (L-Trp), 3 g/L yeast extract, and 1.5 mL/L trace element solution (TES) at pH 6.8. TES is composed of 2.0 g/L Al_2_(SO_4_)_3_∙18H_2_O, 0.75 g/L CoSO_4_∙7H_2_O, 2.5 g/L CuSO_4_∙5H_2_O, 0.5 g/L H_3_BO_3_, 14.64 g/L MnSO_4_∙H_2_O, 3 g/L Na_2_MoO_4_∙2H_2_O, 2.5 g/L NiSO_4_∙6H_2_O, 15 g/L ZnSO_4_∙7H_2_O and 10 mL/L HCl. 12 g/L CaCO_3_ was added as a neutralizing agent to adjust the pH of the medium. For the production of cinnamic acid and cinnamaldehyde, LB medium was used.

Inoculation was performed in LB medium containing 2 % (w/v) glucose at 37 °C with shaking at 200 rpm. After overnight cultivation, 2.5 mL of cells were transferred to 250 mL Erlenmeyer flasks containing 50 mL of medium (5 % (v/v) inoculation). When cell density reached an OD_600_ of mid-exponential phase, isopropyl-β-D-thiogalactopyranoside (IPTG; Sigma–Aldrich, St. Louis, MO, USA) was added to a final concentration of 0.1 mM to induce gene expression. Whenever sampling was required, cells were harvested by centrifugation at 13,000 rpm for 10 min at 4 °C, and the pellet and supernatant were separately stored at −20 °C until further analysis.

### Enzyme purification and analysis

After cultivation of *E. coli* BL21(DE3) harboring the pHB-I series (pHB-I01–pHB-I05), cells were harvested by centrifugation at 6000 rpm for 10 min at 4 °C. Harvested cells were re-suspended in Talon^®^ equilibration buffer (50 mM Na_3_PO_4_, 300 mM NaCl, pH 7.0), and disrupted by sonication (VCX 750, Sonics & Materials. Inc., Newtown, CT, USA) while chilled on ice at 50 % pulse and 20 % amplitude for 30 min. Cell lysates were centrifuged at 10,000 rpm for 10 min at 4 °C, and the soluble fractions were collected from the supernatant and filtered using 0.45 μm filters. Soluble fractions were mixed with Talon metal-affinity resin (Clontech, Mountain View, CA, USA) in poly-prep chromatography columns (Bio-Rad). After the binding of each enzyme, which was tagged with polyhistidine, the resin was washed with 10 mL of wash buffer (equilibration buffer containing 15 mM imidazole), and enzymes were eluted using 3 mL of elution buffer (equilibration buffer containing 150 mM imidazole).

Protein samples were analyzed using 12 % (w/v) SDS–polyacrylamide gel electrophoresis (SDS–PAGE). After gel electrophoresis, gels were stained with Coomassie brilliant blue (50 % (v/v) methanol, 10 % (v/v) acetic acid, and 1 g/L Coomassie brilliant blue R-250) for 30 min and destained using destaining solution (10 % (v/v) methanol and 10 % (v/v) acetic acid). For western blot analysis, electrophoresed protein samples were transferred to polyvinyl difluoride (PVDF; Roche, Basel, Switzerland) membranes using Bio-Rad Trans-blot SD (Bio-Rad) at 70 mA per gel for 90 min. After blocking with 5 % (w/v) skim milk solution in Tris-buffered saline containing Tween-20 (TBS-T; 10 mM Tris, 150 mM NaCl and 0.05 % (v/v) Tween-20, pH 8.0), membranes were incubated with a horseradish peroxidase (HRP)-conjugated anti-histidine antibody or an anti-FLAG antibody (Sigma–Aldrich) dissolved in TBS-T with 5 % (w/v) skim milk. Each step was performed for 1 h at room temperature. After washing with TBS-T four times, ECL western blotting detection reagent (Bionote, Hwaseong, Republic of Korea) was added and signals were detected on X-ray films.

### Quantification of enzyme activity

To test the enzymatic activity of PAL enzymes, enzymes were mixed with 0.2 mM l-phenylalanine and 250 pmol of PAL from *A. thaliana* or *S. maritimus* in 1 mL of 100 mM Tris–HCl (pH 7.5) and incubated at 30 and 37 °C for 1 h [23]. To test the enzymatic activity of 4CL and CCR, 500 pmol of AtCCR and 500 pmol of At4CL1 (or ScCCL) were mixed with 2.5 mM ATP, 2.5 mM MgCl_2_, 0.2 μM CoA, 0.1 mM NADPH, and 0.2 mM *trans*-cinnamic acid in 1 mL of 100 mM Tris–HCl (pH 7.5) and incubated at 30 and 37 °C for 1 h [27]. In the reaction by At4CL1 (or ScCCL), CoA consumed by At4CL1 (or ScCCL) can be regenerated by AtCCR in the next step (Fig. 1a), so a smaller amount of CoA (0.2 μM) was used than the substrate (cinnamic acid). Both cinnamic acid and cinnamaldehyde in the reaction were quantified using reverse-phase high-performance liquid chromatography (HPLC) (Additional file 2: Figure S2).

### Analytical procedures

Cell growth was determined by measuring the optical density at 600 nm (OD_600_) with a spectrophotometer (Optizen POP; Mecasys, Daejeon, Republic of Korea). The concentration of glucose was determined by a glucose analyzer (YSI 2700 SELECT™ Biochemistry Analyzer; YSI Life Sciences, Yellow Springs, OH, USA). Cinnamic acid and cinnamaldehyde were quantified using a HPLC (LC-20AD, CTO-20A, SPD-20A; Shimadzu, Kyoto, Japan) equipped with a Zorbax Eclipse AAA column (4.6 × 150 mm 3.5-Micron; Agilent, Santa Clara, CA, USA). Samples were sterile filtered using 0.22 μm PVDF syringe filters (Futecs Co., Ltd., Daejeon, Republic of Korea). Samples were separated using a binary nonlinear gradient with mobile phase A [0.1 % (v/v) trifluoroacetic acid (TFA)] and mobile phase B (acetonitrile). The column temperature was maintained at 40 °C and the flow rate was 1 mL/min. Elution conditions were as follows: (i) equilibrate with 10 % B for 1 min, (ii) run gradient from 10 to 70 % B for 19 min, (iii) run gradient from 70 to 10 % B for 5 min, and (iv) clean with 10 % B for 3 min. Samples were detected using a UV detector (280 nm). The analysis of l-phenylalanine was performed under nearly identical conditions used for cinnamic acid and cinnamaldehyde analysis except that elution conditions were different. Elutions were performed as follows: (i) equilibrate with 10 % B for 6 min, (ii) run gradient from 10 to 70 % B for 4 min, (iii) maintain flow at 70 % B for 7 min, (iv) run gradient from 70 to 10 % B for 3 min, and (v) wash with 10 % B for 5 min. Samples were detected using a UV detector (220 nm). The standard curves for phenylalanine (0.1–1 g/L), *trans*-cinnamic acid (5–200 mg/L), and cinnamaldehyde (1–150 mg/L) were determined using similar procedures. l-phenylalanine, *trans*-cinnamic acid, and cinnamaldehyde were purchased from Sigma–Aldrich.

### Bioassays of nematicidal activity

To evaluate the nematicidal activity of cinnamaldehyde produced by *E. coli*, direct contact bioassays were performed [12]. Briefly, nematodes were suspended in distilled water at a density of 5000 nematodes/mL, and 15 μL of the suspension (about 75 nematodes) was mixed with 60 μL of the following solutions: culture medium (negative control), commercial cinnamaldehyde dissolved in 10 % (v/v) acetonitrile (positive control), and cinnamaldehyde produced by *E. coli* prepared in this study. After 4 h of treating nematodes with the solutions, the number of nematodes that remained alive was counted using a microscope. If nematodes were stretched and non-motile, they were scored as dead.

## Additional files

10.1186/s12934-016-0415-9 SDS–PAGE analysis of protein purifications.
10.1186/s12934-016-0415-9 Standard curves of cinnamic acid and cinnamaldehyde.
10.1186/s12934-016-0415-9 Gel electrophoresis data for mutant strain verification (YHP01 to YHP05).
10.1186/s12934-016-0415-9 Construction of plasmid-based overexpression system for l-phenylalanine biosynthesis.
10.1186/s12934-016-0415-9 The growth curve and l-phenylalanine titer curve of final engineered strain (YHP05/pYHP) (A) and glucose consumption curve (B).
10.1186/s12934-016-0415-9 Western blot analysis of enzymes produced after cultivations using (A) anti-His-HRP and (B) anti-FLAG-HRP.
10.1186/s12934-016-0415-9 Plasmid construction.
10.1186/s12934-016-0415-9 List of primers used in PCR experiments.
10.1186/s12934-016-0415-9 Designed 5′-UTR sequences and predicted expression level.
10.1186/s12934-016-0415-9 Strain construction.

## Abbreviations

PAL: phenylalanine-ammonia lyase
4CL: 4-coumarate:CoA ligase
CCR: cinnamoyl-CoA reductase
CCL: cinnamate:CoA ligase
AAA: aromatic amino acid
PTS: phosphotransferase system
PEP: phosphoenolpyruvate
DAHP: 3-deoxy-d-arabinoheptulosonate 7-phosphate
PheA^fbr,dm^: feedback-resistant chorismate mutase/prephenate dehydratase with double mutations

## References

[1] Jones JT, Haegeman A, Danchin EGJ, Gaur HS, Helder J, Jones MGK, Kikuchi T, Manzanilla-Lopez R, Palomares-Rius JE, Wesemael WML, Perry RN (2013). Top 10 plant-parasitic nematodes in molecular plant pathology. Mol Plant Pathol.

[2] Wesemael WML, Viaene N, Moens M (2011). Root-knot nematodes (*Meloidogyne* spp.) in Europe. Nematology.

[3] Jabbar A, Ali MA, Khan SA, Javed N (2015). Root-knot nematodes an emerging threat to cereal crops. J Green Physiol Genet Genom.

[4] Haegeman A, Mantelin S, Jones JT, Gheysen G (2012). Functional roles of effectors of plant-parasitic nematodes. Gene.

[5] McCarter JP, Mitreva MD, Martin J, Dante M, Wylie T, Rao U, Pape D, Bowers Y, Theising B, Murphy CV, Kloek AP, Chiapelli BJ, Clifton SW, Bird DM, Waterston RH (2003). Analysis and functional classification of transcripts from the nematode *Meloidogyne incognita*. Genome Biol.

[6] Li W, Wang K, Chen L, Johnson JA, Wang S (2015). Tolerance of *Sitophilus zeamais* (Coleoptera: Curculionidae) to heated controlled atmosphere treatments. J Stored Prod Res.

[7] Thomson LJ, Hoffmann AA (2010). Natural enemy responses and pest control: importance of local vegetation. Biol Control.

[8] Giannakou IO, Karpouzas DG (2003). Evaluation of chemical and integrated strategies as alternatives to methyl bromide for the control of root-knot nematodes in Greece. Pest Manag Sci.

[9] Wang G, Chen X, Deng Y, Li Z, Xu X (2015). Synthesis and nematicidal activities of 1,2,3-benzotriazin-4-one derivatives against *Meloidogyne incognita*. J Agric Food Chem.

[10] Bock CH, Shapiro-Ilan DI, Wedge DE, Cantrell CL (2014). Identification of the antifungal compound, trans-cinnamic acid, produced by *Photorhabdus luminescens*, a potential biopesticide against pecan scab. J Pest Sci.

[11] Ntalli NG, Caboni P (2012). Botanical nematicides: a review. J Agric Food Chem.

[12] Kong JO, Lee SM, Moon YS, Lee SG, Ahn YJ (2007). Nematicidal activity of cassia and cinnamon oil compounds and related compounds toward *Bursaphelenchus xylophilus* (Nematoda: Parasitaphelenchidae). J Nematol.

[13] Ooi LSM, Li Y, Kam SL, Wang H, Wong EYL, Ooi VEC (2006). Antimicrobial activities of cinnamon oil and cinnamaldehyde from the Chinese medicinal herb *Cinnamomum cassia* Blume. Am J Chin Med.

[14] Bevilacqua A, Corbo MR, Sinigaglia M (2008). Combined effects of low pH and cinnamaldehyde on the inhibition of *Alicyclobacillus acidoterrestris* spores in a laboratory medium. J Food Process Pres.

[15] Muhammad JS, Zaidi SF, Shaharyar S, Refaat A, Usmanghani K, Saiki I, Sugiyama T (2015). Anti-inflammatory effect of cinnamaldehyde in *Helicobacter pylori* induced gastric inflammation. Biol Pharm Bull.

[16] Lungarini S, Aureli F, Coni E (2008). Coumarin and cinnamaldehyde in cinnamon marketed in Italy: a natural chemical hazard?. Food addit Contam Part A.

[17] Hsu KH, Huang WK, Lin YL, Chang ST, Chu FH (2012). A genetic marker of 4-coumarate:coenzyme A ligase gene in the cinnamaldehyde-chemotype *Cinnamomum osmophloeum*. Holzforschung.

[18] da Cunha A (1988). Purification, characterization and induction of l-phenylalanine ammonia-lyase in *Phaseolus vulgaris*. Eur J Biochem.

[19] Ehlting J, Buttner D, Wang Q, Douglas CJ, Somssich IE, Kombrink E (1999). Three 4-coumarate:coenzyme A ligases in *Arabidopsis thaliana* represent two evolutionarily divergent classes in angiosperms. Plant J.

[20] Wengenmayer H, Ebel J, Grisebach H (1976). Enzymic synthesis of lignin precursors. Purification and properties of a cinnamoyl-CoA: NADPH reductase from cell suspension cultures of soybean (*Glycine max*). Eur J Biochem.

[21] Cui JD, Qiu JQ, Fan XW, Jia SR, Tan ZL (2014). Biotechnological production and applications of microbial phenylalanine ammonia lyase: a recent review. Crit Rev Biotechnol.

[22] Cochrane FC, Davin LB, Lewis NG (2004). The *Arabidopsis* phenylalanine ammonia lyase gene family: kinetic characterization of the four PAL isoforms. Phytochemistry.

[23] Xiang L, Moore BS (2005). Biochemical characterization of a prokaryotic phenylalanine ammonia lyase. J Bacteriol.

[24] Hamberger B, Hahlbrock K (2004). The 4-coumarate:CoA ligase gene family in *Arabidopsis thaliana* comprises one rare, sinapate-activating and three commonly occurring isoenzymes. Proc Natl Acad Sci USA.

[25] Costa MA, Bedgar DL, Moinuddin SGA, Kim KW, Cardenas CL, Cochrane FC, Shockey JM, Helms GL, Amakura Y, Takahashi H, Milhollan JK, Davin LB, Browse J, Lewis NG (2005). Characterization in vitro and in vivo of the putative multigene 4-coumarate:CoA ligase network in *Arabidopsis*: syringyl lignin and sinapate/sinapyl alcohol derivative formation. Phytochemistry.

[26] Baltas M, Lapeyre C, Bedos-Belval F, Maturano M, Saint-Aguet P, Roussel L, Duran H, Grima-Pettenati J (2005). Kinetic and inhibition studies of cinnamoyl-CoA reductase 1 from *Arabidopsis thaliana*. Plant Physiol Biochem.

[27] Kaneko M, Ohnishi Y, Horinouchi S (2003). Cinnamate: coenzyme A ligase from the filamentous bacterium *Streptomyces coelicolor* A3(2). J Bacteriol.

[28] Deutscher J, Francke C, Postma PW (2006). How phosphotransferase system-related protein phosphorylation regulates carbohydrate metabolism in bacteria. Microbiol Mol Biol Rev.

[29] Gosset G (2005). Improvement of *Escherichia coli* production strains by modification of the phosphoenolpyruvate: sugar phosphotransferase system. Microb Cell Fact.

[30] Pittard J, Camakaris H, Yang J (2005). The TyrR regulon. Mol Microbiol.

[31] Gosset G (2009). Production of aromatic compounds in bacteria. Curr Opin Biotechnol.

[32] Rodriguez A, Martinez JA, Flores N, Escalante A, Gosset G, Bolivar F (2014). Engineering *Escherichia coli* to overproduce aromatic amino acids and derived compounds. Microb Cell Fact.

[33] Meza E, Becker J, Bolivar F, Gosset G, Wittmann C (2012). Consequences of phosphoenolpyruvate: sugar phosphotransferase system and pyruvate kinase isozymes inactivation in central carbon metabolism flux distribution in *Escherichia coli*. Microb Cell Fact.

[34] Sprenger GA (2007). From scratch to value: engineering *Escherichia coli* wild type cells to the production of l-phenylalanine and other fine chemicals derived from chorismate. Appl Microbiol Biotechnol.

[35] Ding R, Liu L, Chen X, Cui Z, Zhang A, Ren D, Zhang L (2014). Introduction of two mutations into AroG increases phenylalanine production in *Escherichia coli*. Biotechnol Lett.

[36] Dell KA, Frost JW (1993). Identification and removal of impediments to biocatalytic synthesis of aromatics from d-glucose: rate-limiting enzymes in the common pathway of aromatic amino acid biosynthesis. J Am Chem Soc.

[37] Lutke-Eversloh T, Stephanopoulos G (2008). Combinatorial pathway analysis for improved l-tyrosine production in *Escherichia coli*: identification of enzymatic bottlenecks by systematic gene overexpression. Metab Eng.

[38] Zhang S, Pohnert G, Kongsaeree P, Wilson DB, Clardy J, Ganem B (1998). Chorismate mutase-prephenate dehydratase from *Escherichia coli*. Study of catalytic and regulatory domains using genetically engineered proteins. J Biol Chem.

[39] Zhang S, Wilson DB, Ganem B (2000). Probing the catalytic mechanism of prephenate dehydratase by site-directed mutagenesis of the *Escherichia coli* P-protein dehydratase domain. Biochemistry.

[40] Yi J, Draths KM, Li K, Frost JW (2003). Altered glucose transport and shikimate pathway product yields in *E. coli*. Biotechnol Prog.

[41] Liu SP, Liu RX, Xiao MR, Zhang L, Ding ZY, Gu ZH, Shi GY (2014). A systems level engineered *E. coli* capable of efficiently producing l-phenylalanine. Process Biochem.

[42] Vannelli T, Wei Qi W, Sweigard J, Gatenby AA, Sariaslani FS (2007). Production of *p*-hydroxycinnamic acid from glucose in *Saccharomyces cerevisiae* and *Escherichia coli* by expression of heterologous genes from plants and fungi. Metab Eng.

[43] Vargas-Tah A, Martinez LM, Hernandez-Chavez G, Rocha M, Martinez A, Bolivar F, Gosset G (2015). Production of cinnamic acid and p-hydroxycinnamic acid from sugar mixtures with engineered *Escherichia coli*. Microb Cell Fact.

[44] Zhou H, Liao X, Liu L, Wang T, Du G, Chen J (2011). Enhanced l-phenylalanine production by recombinant *Escherichia coli* BR-42 (pAP-B03) resistant to bacteriophage BP-1 via a two-stage feeding approach. J Ind Microbiol Biotechnol.

[45] Yim SC, Jeong KJ, Chang HN, Lee SY (2001). High-level secretory production of human granulocyte-colony stimulating factor by fed-batch culture of recombinant *Escherichia coli*. Bioprocess Biosyst Eng.

[46] Jeong KJ, Lee SY (2003). Enhanced production of recombinant proteins in *Escherichia coli* by filamentation suppression. Appl Environ Microbiol.

[47] Dueber JE, Wu GC, Malmirchegini GR, Moon TS, Petzold CJ, Ullal AV, Prather KLJ, Keasling JD (2009). Synthetic protein scaffolds provide modular control over metabolic flux. Nat Biotechnol.

[48] Lee JH, Jung SC, Bui LM, Kang KH, Song JJ, Kim SC (2013). Improved production of l-threonine in *Escherichia coli* by use of a DNA scaffold system. Appl Environ Microbiol.

[49] Sambrook J, Russell DW (2001). Molecular cloning: a laboratory manual. Q Rev Biol.

[50] Song CW, Lee SY (2013). Rapid one-step inactivation of single or multiple genes in *Escherichia coli*. Biotechnol J.

[51] Gerigk MR, Maass D, Kreutzer A, Sprenger G, Bongaerts J, Wubbolts M, Takors R (2002). Enhanced pilot-scale fed-batch l-phenylalanine production with recombinant *Escherichia coli* by fully integrated reactive extraction. Bioprocess Biosyst Eng.

[52] Yakandawala N, Romeo T, Friesen AD, Madhyastha S (2008). Metabolic engineering of *Escherichia coli* to enhance phenylalanine production. Appl Microbiol Biotechnol.

[53] Yim SS, An SJ, Kang M, Lee J, Jeong KJ (2013). Isolation of fully synthetic promoters for high-level gene expression in *Corynebacterium glutamicum*. Biotechnol Bioeng.

[54] Qian ZG, Xia XX, Lee SY (2009). Metabolic engineering of *Escherichia coli* for the production of putrescine: a four carbon diamine. Biotechnol Bioeng.

[55] Kim JM, Lee KH, Lee SY (2008). Development of a markerless gene knock-out system for *Mannheimia succiniciproducens* using a temperature-sensitive plasmid. FEMS Microbiol Lett.

