# Supplementary material for: Metabolic engineering of Escherichia coli for the production of cinnamaldehyde
Source: Microb Cell Fact. 2016 Jan 19;15:16. doi: 10.1186/s12934-016-0415-9 (PMC4719340; doi:10.1186/s12934-016-0415-9)
Supplement: Supplementary file 1 — 10.1186/s12934-016-0415-9 SDS–PAGE analysis of protein purifications. [file 12934_2016_415_MOESM1_ESM.pdf]

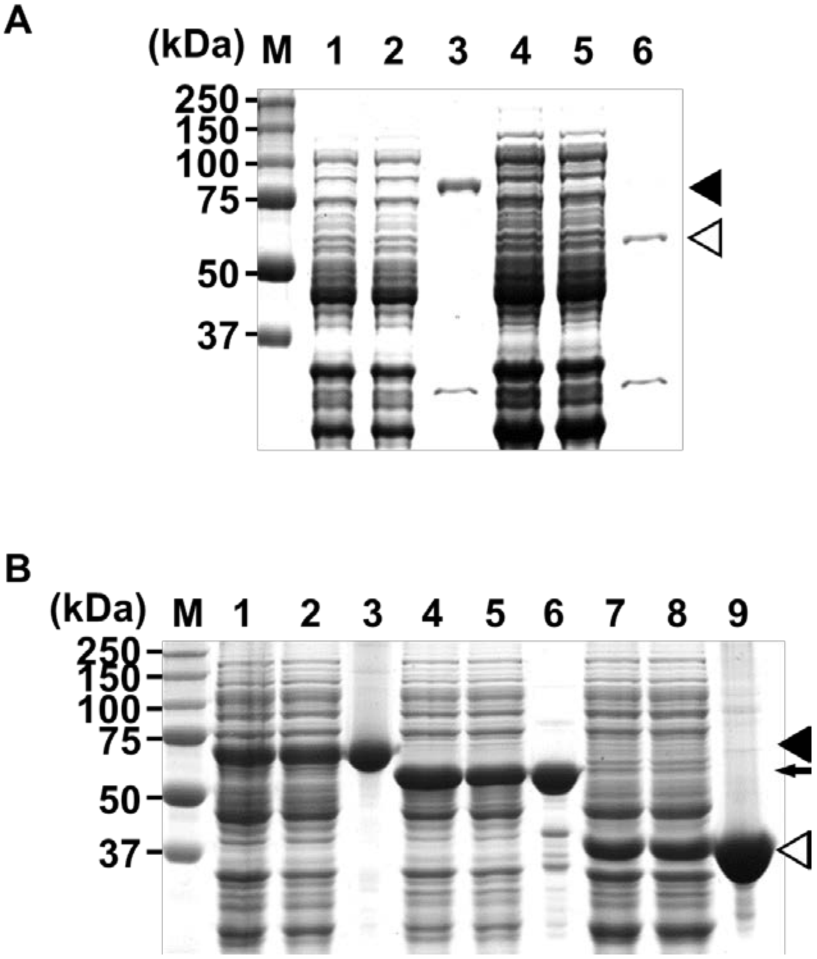

Additional file 1: Figure S1. SDS-PAGE analysis of protein purifications. (A) Purification of PAL. Lane M, molecular weight size marker (kDa); Lanes 1, 2, and 3, total, soluble, and elution fraction of AtPAL1 (closed arrowhead, ◀), respectively; lanes 4, 5, and 6, total, soluble, and elution fraction of SmPAL (open arrowhead, ◁), respectively. (B) Purification of At4CL1, ScCCL, and AtCCR. Lane M, molecular weight size marker (kDa); Lanes 1, 2, and 3, total, soluble, and elution fraction of At4CL1 (closed arrowhead, ◀), respectively; lanes 4, 5, and 6, total, soluble, and elution fraction of ScCCL (solid arrow, ←), respectively; lanes 7, 8, and 9, total, soluble, and elution fraction of AtCCR (open arrowhead, ◁), repectively.
