# Supplementary material for: Metabolic engineering of Escherichia coli for the production of cinnamaldehyde
Source: Microb Cell Fact. 2016 Jan 19;15:16. doi: 10.1186/s12934-016-0415-9 (PMC4719340; doi:10.1186/s12934-016-0415-9)
Supplement: Supplementary file 2 — 10.1186/s12934-016-0415-9 Standard curves of cinnamic acid and cinnamaldehyde. [file 12934_2016_415_MOESM2_ESM.pdf]

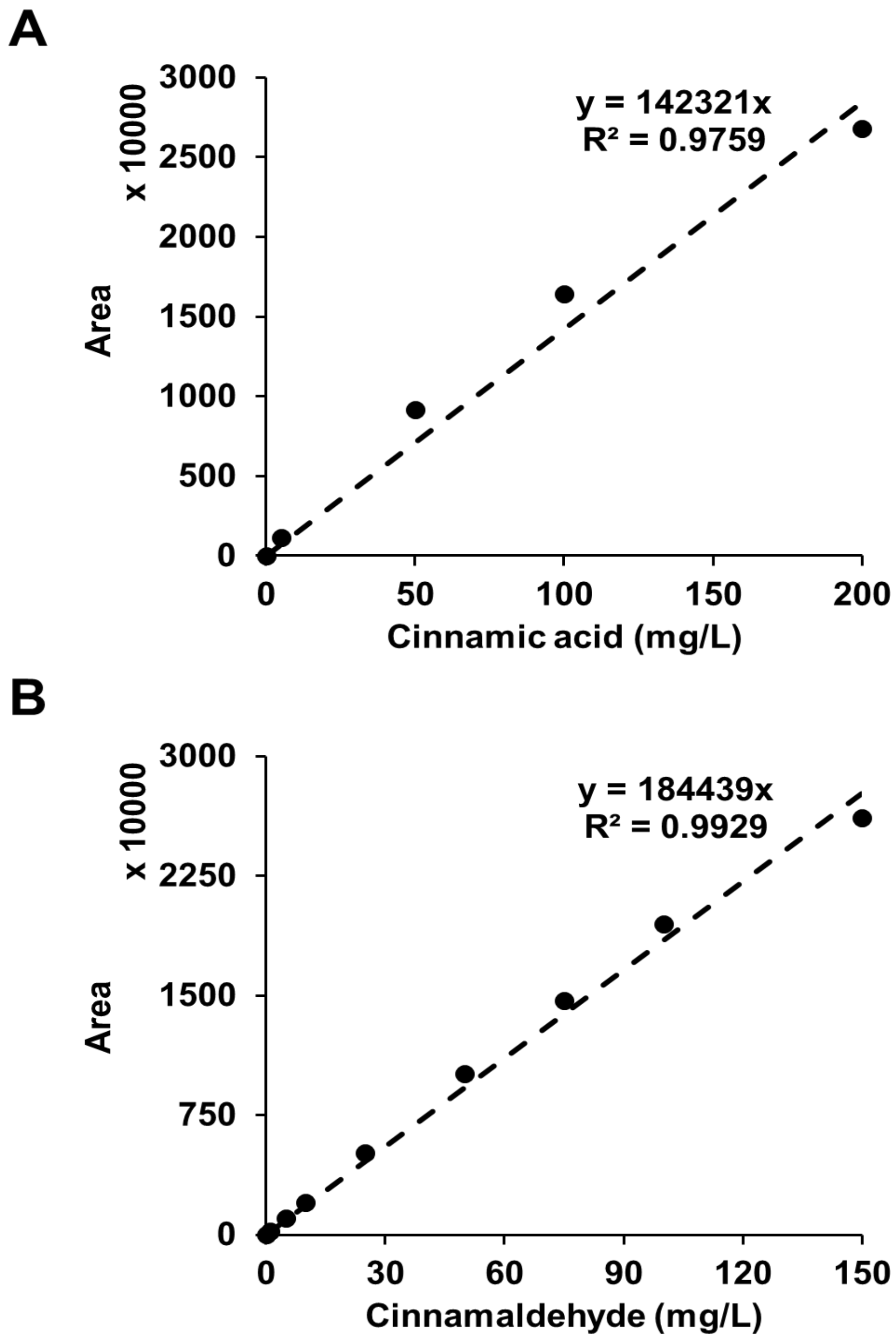

Additional file 2: Figure S2. Standard curves of cinnamic acid and cinnamaldehyde. (A) Standard curve of cinnamic acid by using the concentrations of cinnamic acid solutions (5, 50, 100, 200 mg/L). (B) Standard curve of cinnamaldehyde by using the concentrations of cinnamaldehyde solutions (1, 5, 10, 25, 50, 75, 100, 150 mg/L).
