# Supplementary material for: Metabolic engineering of Escherichia coli for the production of cinnamaldehyde
Source: Microb Cell Fact. 2016 Jan 19;15:16. doi: 10.1186/s12934-016-0415-9 (PMC4719340; doi:10.1186/s12934-016-0415-9)
Supplement: Supplementary file 3 — 10.1186/s12934-016-0415-9 Gel electrophoresis data for mutant strain verification (YHP01 to YHP05). [file 12934_2016_415_MOESM3_ESM.pdf]

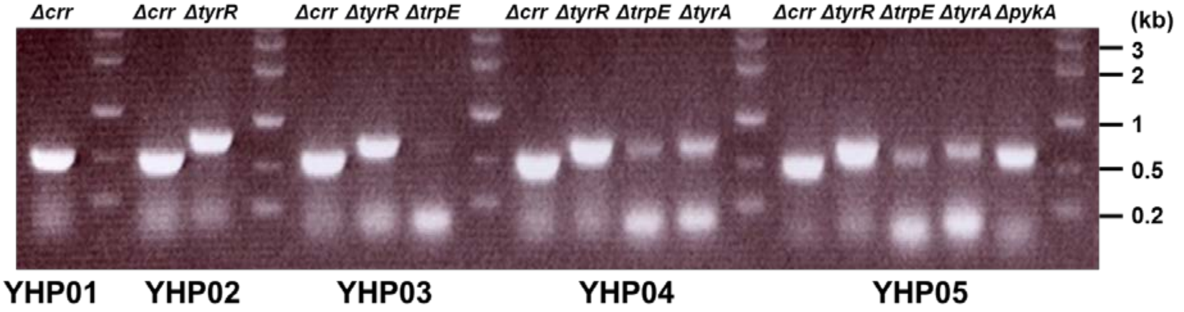

Additional file 3: Figure S3. Gel electrophoresis data for mutant strain verification (YHP01 to YHP05). Each lane indicates the genomic DNA PCR products of each labeled genes.
