# Supplementary material for: Metabolic engineering of Escherichia coli for the production of cinnamaldehyde
Source: Microb Cell Fact. 2016 Jan 19;15:16. doi: 10.1186/s12934-016-0415-9 (PMC4719340; doi:10.1186/s12934-016-0415-9)
Supplement: Supplementary file 4 — 10.1186/s12934-016-0415-9 Construction of plasmid-based overexpression system for l-phenylalanine biosynthesis. [file 12934_2016_415_MOESM4_ESM.pdf]

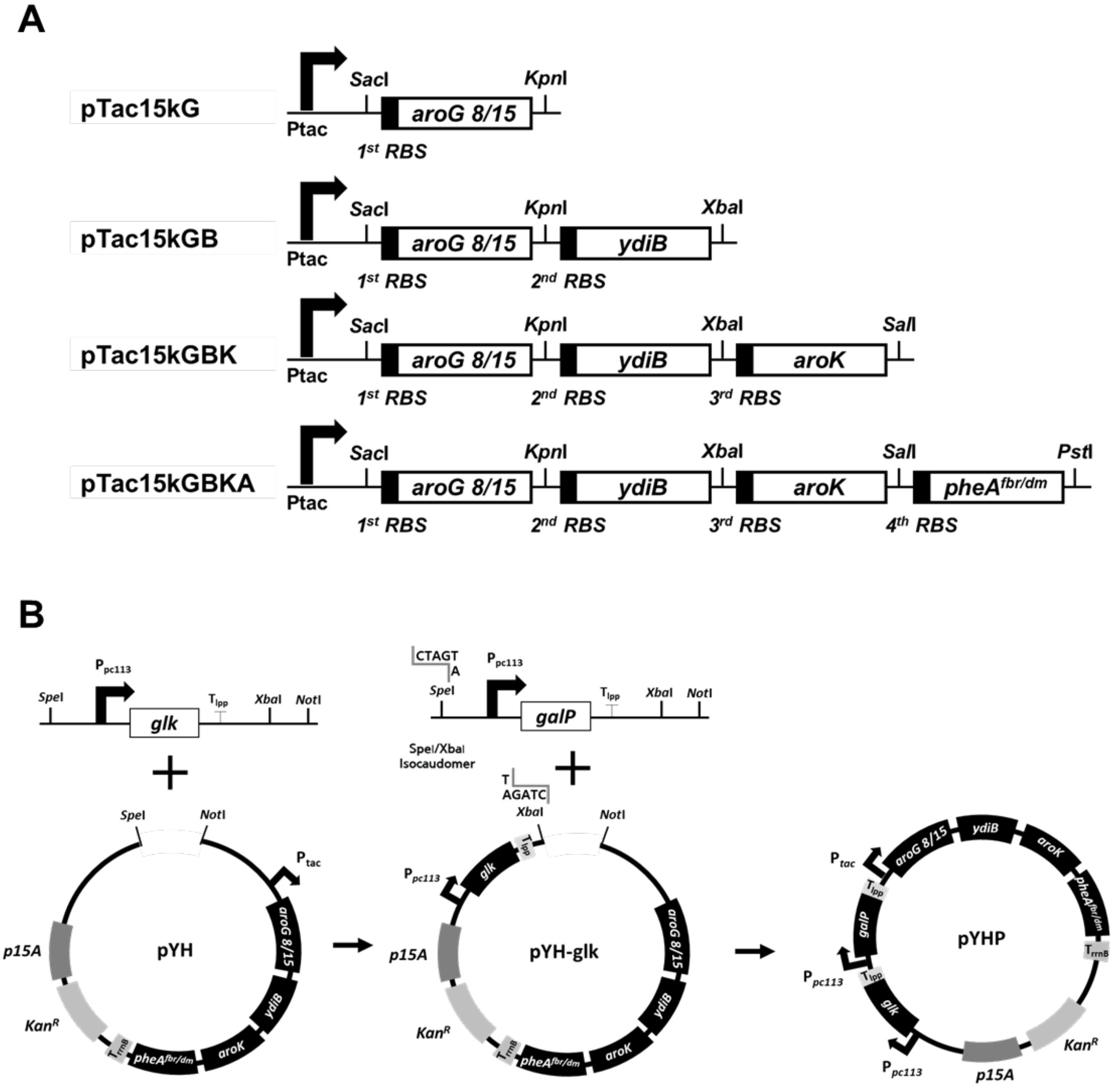

Additional file 4: Figure S4. Construction of plasmid-based overexpression system for L-phenylalanine biosynthesis. (A) Schematic diagram of polycistronic system was described in regular sequence; *aroG8/15, ydiB, aroK,* $pheA^{fbr,dm}$ (to generate pTac15kG, pTac15kGB, pTac15kGBK, pTac15kGBKA plasmids) and, (B) schematic diagram of cloning procedure to generate pYHP plasmid by introduction of *galP* and *glk*.
