# Supplementary material for: Metabolic engineering of Escherichia coli for the production of cinnamaldehyde
Source: Microb Cell Fact. 2016 Jan 19;15:16. doi: 10.1186/s12934-016-0415-9 (PMC4719340; doi:10.1186/s12934-016-0415-9)
Supplement: Supplementary file 5 — 10.1186/s12934-016-0415-9 The growth curve and l-phenylalanine titer curve of final engineered strain (YHP05/pYHP) (A) and glucose consumption curve (B). [file 12934_2016_415_MOESM5_ESM.pdf]

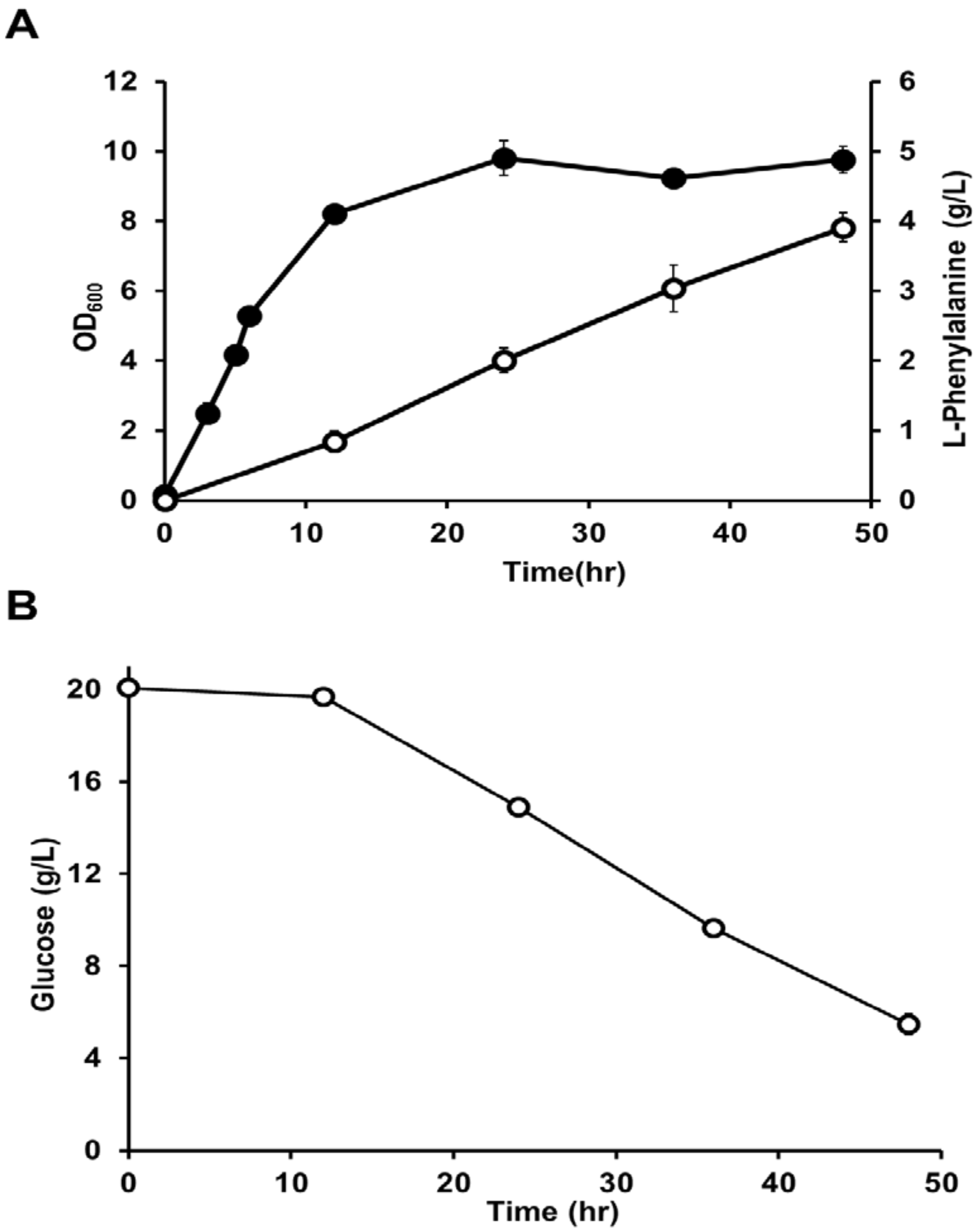

Additional file 5: Figure S5. The growth curve and L-phenylalanine titer curve of final engineered strain (YHP05/pYHP) (A) and glucose consumption curve (B). (A) Symbols: Closed circle (●), growth curve; open circle (○), phenylalanine titer (g/L). (B) Symbols: open circle (○), *E. coli* YHP05 harboring pYHP. Error bars represent standard deviation of the mean and n = 3
