# Supplementary material for: Metabolic engineering of Escherichia coli for the production of cinnamaldehyde
Source: Microb Cell Fact. 2016 Jan 19;15:16. doi: 10.1186/s12934-016-0415-9 (PMC4719340; doi:10.1186/s12934-016-0415-9)
Supplement: Supplementary file 6 — 10.1186/s12934-016-0415-9 Western blot analysis of enzymes produced after cultivations using (A) anti-His-HRP and (B) anti-FLAG-HRP. [file 12934_2016_415_MOESM6_ESM.pdf]

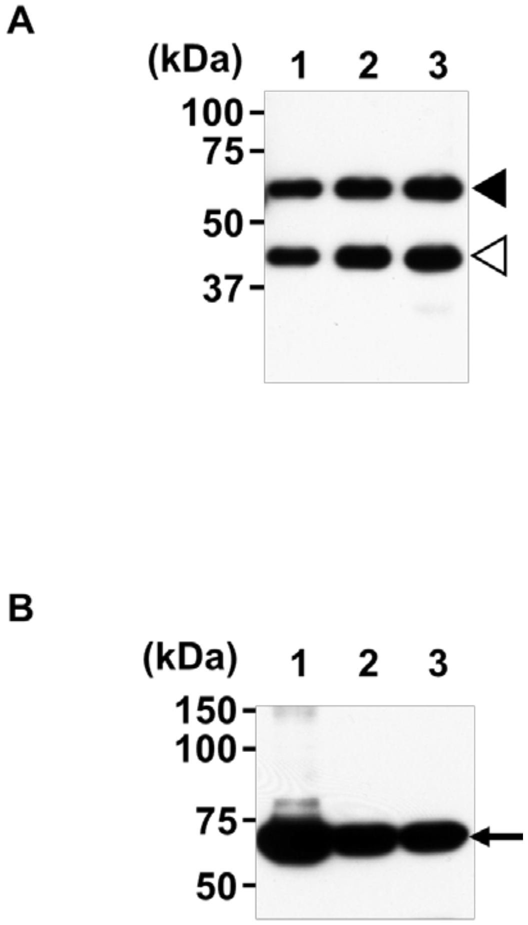

Additional file 6: Figure S6. Western blot analysis of enzymes produced after cultivations using (A) anti-His-HRP and (B) anti-FLAG-HRP. Lanes 1, 2, and 3, soluble fraction from *E. coli* W3110 harboring pHB-CAD, *E. coli* YHP05 harboring pHB-CAD, and *E. coli* YHP05 harboring pHB-CAD and pYHP. Symbols: Closed arrowhead (◀), ScCCL; open arrowhead (◁), AtCCR; solid arrow (⟵), SmPAL.
