# Supplementary material for: Metabolic engineering of Escherichia coli for the production of cinnamaldehyde
Source: Microb Cell Fact. 2016 Jan 19;15:16. doi: 10.1186/s12934-016-0415-9 (PMC4719340; doi:10.1186/s12934-016-0415-9)
Supplement: Supplementary file 7 — 10.1186/s12934-016-0415-9 Plasmid construction. [file 12934_2016_415_MOESM7_ESM.docx]

**Additional file 7: Plasmid construction**

**1.** pHB-CA, and pHB-CAD, all structural genes were amplified by polymerase chain reaction (PCR) with primers (Additional file 9: Table S1). The cDNA of *Arabidopsis thaliana* was used as a template for gene amplifications of phenylalanine-ammonia lyase (AtPAL1), 4-coumarate: CoA ligase (At4CL1), and cinnamoyl-CoA reductase (AtCCR). To obtain cDNA of *A. thaliana*, seed was frozen in liquid nitrogen before being ground. After grinding, total RNA was extracted by using GenElute^TM^ Total RNA Purification Kit (Sigma-Aldrich), and cDNA of *A. thaliana* was synthesized by reverse-transcription PCR (RT-PCR) using AccessQuick^TM^ RT-PCR System (Promega corp., Fitchburg, WI, USA). The PAL gene of *Streptomyces maritimus* (SmPAL) was synthesized by GenScript (Piscataway, NJ, USA). Cinnamate-CoA ligase of *Streptomyces coelicolor* (ScCCL) was amplified by PCR from genomic DNA. Above genes were amplified with following primers, respectively: AtPAL (P1/P2), At4CL (P3/P4), AtCCR (P5/P6), SmPAL (P7/P8), and ScCCL (P9/P10). Then, all PCR products were digested with restriction enzyme *Xba*I/*Not*I and cloned into pET-22b at the same restriction enzymes sites, yielding pHB-I01 to pHB-I05.

**2.** In case of pHB-CA and pHB-CAD construction, SmPAL, ScCCL, and AtCCR genes were amplified with primers P11/P12, P13/P14, and P15/P16, followed by treatment of restriction enzyme *Nco*I/*Eco*RI, *Eco*RI/*Xba*I, and *Not*I/*Xba*I, respectively. Then, SmPAL gene was ligated into pTrc99A for pHB-CA construction, or all genes were sequentially cloned into pTrc99A for pHB-CAD construction. The sequences used in this study has been deposited in the GenBank data. (AtPAL (AAC18870.1), At4CL (AEE32700.1), AtCCR (AEE36454.1), SmPAL (AAF81735.1), ScCCL (CAB95894.1).

**3.** For the construction of pYHP plasmid, all structural genes were amplified by PCR. The polycistronic expression system under the strong *tac* promoter was constructed including *aroG8/15* (3-deoxy-d-arabinoheptulosonate 7-phosphate (DAHP) mutated with D146N and A202T), *ydiB* (shikimate dehydrogenase)*, aroK* (shikimate kinase)*, pheA^fbr,dm^* (feedback-resistant chorismate mutase/prephenate dehydratase mutated with E159A and E232A). The *aroG8/15* and *pheA^fbr,dm^* were amplified by site directed mutagenesis PCR. The *ydiB* and *aroK* genes were amplified by conventional PCR from the genomic DNA of *E. coli* K12 strain as a template. Four structural genes were sub-cloned into pUC19 vector. To make the aroG8/15 mutant, D146N, A202T mutations were introduced in the wild type *aroG* gene. The *aroG8/15* gene was amplified by overlapping PCR using the primer combinations P17/P21 (Frag1), P20/P19 (Frag2) and P18/P22 (Frag3), respectively. Frag1, Frag2 and Frag3 have the desired point mutations (D146N, A202T) and overlapping sequence with another fragment. Thus, the three PCR fragments could be combined to the one fragment using the primer combination P17/P22 (PCR1). The PCR1 product was digested with *Sac*I*/Kpn*I and ligated with pUC19 vector to generate pUC19G. The *ydiB* gene was amplified by PCR using the primer combination P23/P24 (PCR2). The PCR2 product was digested with *Kpn*I*/Xba*I and ligated with pUC19 to generate pUC19B. The *aroK* gene was amplified by PCR using the primer combination P25/P26 (PCR3). The PCR3 product was digested with *Xba*I*/Sal*I and ligated with pUC19 to generate pUC19K. In the wild type *pheA* gene, E159A, E232A mutations were introduced. The *pheA^(fbr,dm)^* gene was amplified by overlapping PCR using the primer combinations P27/P29 (Frag4), P28/P31 (Frag5) and P30/P32 (Frag6), respectively. Frag4, Frag5 and Frag6 have the desired point mutations (E159A, E232A) and overlapping sequence with another fragment. Thus, the three PCR fragments could be combined to on fragment using the primer combination P27/P32 (PCR4). The PCR4 product was digested with *Sal*I*/Pst*I and ligated with pUC19 vector to generate pUC19A. In the case of construction for polycistronic system, all 5*'*-UTR regions for each genes were designed by UTR designer tool and the 5*'*-UTR regions were extended in front of each genes by using P17, P23, P25 and P27 primers, respectively. The details about designed UTR are described in Additional file 10. Subsequent to the sub-cloning steps, each sub-cloned gene (pUC19G, pUC19B, pUC19K, pUC19A) was double digested with proper restriction enzymes corresponding to the described enzyme sites set in above information. And then ligated them into one polycistronic system in regular sequence; *aroG8/15, ydiB, aroK, pheA^fbr,dm^* to generate pTac15kG, pTac15kGB, pTac15kGBK, pTac15kGBKA plasmids, respectively.

**4.** We introduced *glk* (glucokinase) and *galP* (galactose permease) genes which are related to glucose uptake and phosphorylation. For construction of pYHP, synthetic BBa_J23113 Anderson constitutive promoter (P*pc113*; http://parts.igem.org/) was self-assembled by using the complementary primer combination P35/P36. This fragment has the sticky end of *Xba*I and *Hind*III enzyme site. P*pc113* fragment was digested with *Xba*I*/Hind*III and sub-cloned into pUC19 vector to generate pPpc113. And then, *glk* and *galP* genes were amplified by using the primer combinations P38/P39 (Frag7), P40/P41 (Frag8), respectively. Frag7 and Frag8 have the overlapping sequence of P*pc113* and lpp terminator. Subsequent to making Frag7 and Frag8, P*pc113* and *Spe*I*/Not*I enzyme sites were extended by PCR using the primer combination P37/P42 with Ppc113 fragment, Frag7 and Frag8 as a template. The PCR products were digested with *Spe*I*/Not*I and ligated with pTrc99a-mod to generate pGlk and pGalP plasmids.

**5.** In order to introduce *glk* and *galP* genes into pTac15kGBKA, we modified pTac15kGBKA plasmid due to lack of suitable enzyme sites for cloning. The insertion fragment for the modification was self-assembled by using the complementary primer combination P33/P34. We chose the *Sac*II enzyme site as a modification target site, and the fragment including *Spe*I*/Not*I enzyme site was cloned into *Sac*II site of pTac15kGBKA to generate pYH.

**6.** For cloning the *glk* gene, P*pc113*-*glk*-T_lpp_ fragment was digested with *Spe*I*/Not*I and ligated with pYH to generate pYH-glk. And then, in the case of cloning the *galP*, Ppc113-*galP*-T_lpp_ fragment was also digested with *Spe*I*/Not*I. On the other hand, pYH-*glk* vector was digested with *Xba*I*/Not*I. Subsequent to digestion of insert and vector, pYHP plasmid was obtained by ligation. If the *Xba*I and *Spe*I sites were ligated, the restriction enzyme site would be disappeared. This makes the other genes easily introduce into pYHP vector for further engineering.
