# Supplementary material for: Metabolic engineering of Escherichia coli for the production of cinnamaldehyde
Source: Microb Cell Fact. 2016 Jan 19;15:16. doi: 10.1186/s12934-016-0415-9 (PMC4719340; doi:10.1186/s12934-016-0415-9)
Supplement: Supplementary file 8 — 10.1186/s12934-016-0415-9 List of primers used in PCR experiments. [file 12934_2016_415_MOESM8_ESM.docx]

**Additional file 8: Table S1. List of primers used in PCR experiments**

| No | Sequence^a^ (5*'*→3*'*) | Description |
| --- | --- | --- |
| 1 | GCA**TCTAGA**CACCTTAAGGAGGTCTATCTTTCATATGCACCACCACCACCACCACCACCACATGGAGATTAACGGGGCAC | *AtPAL1* |
| 2 | ATGC**GCGGCCGC**TTATCAACATATTGGAATGGGAGCTCC | *AtPAL1* |
| 3 | GCA**TCTAGA**CCGAAATCAAAAGGAACACCAACGTATGCACCACCACCACCACCACCACCACATGGCGCCACAAGAACAAG | *At4CL1* |
| 4 | ATGC**GCGGCCGC**TTATCACAATCCATTTGCTAGTTTTGCCC | *At4CL1* |
| 5 | GCA**TCTAGA**AACACAAACAAGGAAGGAAGATAAATGCACCACCACCACCACCACCACCACATGCCAGTCGACGTAGCC | *AtCCR* |
| 6 | ATGC**GCGGCCGC**TTATCAAGACCCGATCTTAATGCCATTTTC | *AtCCR* |
| 7 | GCAT**TCTAGA**CCCAACGAAGGGGGAACCACACAATATGCACCACCACCACCACCACCACCACACCTTCGTTATTGAACTGGATATGAATGTTACCC | *SmPAL* |
| 8 | ATGC**GCGGCCGC**TTATCAGTGTGCTGCCACGGCTG | *SmPAL* |
| 9 | GCA**TCTAGA**CGAATACCTGGAGGACCTAAACAGTATGCACCACCACCACCACCACCACCACATGTTCCGCAGCGAGTAC | *ScCCL* |
| 10 | ATGC**GCGGCCGC**TTATCATCGCGGCTCCCTGAGCT | *ScCCL* |
| 11 | GCAT**CCATGG**GGACCTTCGTTATTGAACTGGATATGAATGTTACCC | (poly) *SmPAL* |
| 12 | ATGC**GAATTC**TTATCACTTGTCATCGTCATCCTTGTAGTCGTGTGCTGCCACGGCTG | (poly) *SmPAL* |
| 13 | GCAT**GAATTC**CGAATACCTGGAGGACCTAAACAGTATGCACCACCACCACCACCACCACCACATGTTCCGCAGCGAGTAC | (poly) *ScCCL* |
| 14 | ATGC**TCTAGA**TGC**GCGGCCGC**TTATCATCGCGGCTCCCTGAGCT | (poly) *ScCCL* |
| 15 | GCAT**GCGGCCGC**AAACACAAACAAGGAAGGAAGATAAATGCACCACCACCACCACCACCACCACATGCCAGTCGACGTAGCC | (poly) *AtCCR* |
| 16 | ATGC**TCTAGA**TTATCAAGACCCGATCTTAATGCCATTTTC | (poly) *AtCCR* |
| 17 | ATGCAT**GAGCTC**CGACAAAAAGAAAGGAGCATCTAACATGAATTATCAGAACGACGATTTACG | *aroG*8/15 |
| 18 | AAGTGGCTATCGATGCCATTAATaCCGCCGGTGCGC | *aroG*8 |
| 19 | GCGCACCGGCGGtATTAATGGCATCGATAGCCACTT | *aroG*8 |
| 20 | GCGGCAGGTGAGTTTCTCaATATGATCACCCCACAATATCTCG | *aroG*15 |
| 21 | CGAGATATTGTGGGGTGATCATATtGAGAAACTCACCTGCCGC | *aroG*15 |
| 22 | ATGCAT**GGTACC**TTACCCGCGACGCG | *aroG*8/15 |
| 23 | ATGCAT**GGTACC**AAAACCCCACAAAGGAGCATCAAAGATGGATGTTACCGCAAAATACG | *ydiB* |
| 24 | ATGCAT**TCTAGA**TCAGGCACCGAACCC | *ydiB* |
| 25 | ATGCAT**TCTAGA**AAAAAACAGAAAAGGAGCATCGTGGATGGCAGAGAAACGCAATATC | *aroK* |
| 26 | ATGCAT**GTCGAC**TTAGTTGCTTTCCAGCATGTGA | *aroK* |
| 27 | ATGCAT**GTCGAC**AAAAAAGAAAAAAGGAGCATCTTTGATGACATCGGAAAACCCGTTA | pheA^fbr,dm^ |
| 28 | TGCCGTCGTACCGATTGcAAATACCAGCTCCGGTGC | pheA^fbr,dm^ |
| 29 | GCACCGGAGCTGGTATTTgCAATCGGTACGACGGCA | pheA^fbr,dm^ |
| 30 | CGCACTGGAAGATTGAATATACCGcAAGTACGTCTGCGGCAATG | pheA^fbr,dm^ |
| 31 | CATTGCCGCAGACGTACTTgCGGTATATTCAATCTTCCAGTGCG | pheA^fbr,dm^ |
| 32 | ATGCAT**CTGCAG**TCACAACGTGGTTTTCGCC | pheA^fbr,dm^ |
| 33 | GG**ACTAGT**AATCGATCGATCGATCG**GCGGCCGC**CCGC | pTac15k modify |
| 34 | GG**GCGGCCGC**CGATCGATCGATCGATT**ACTAGT**CCGC | pTac15k modify |
| 35 | CTAGACTGATGGCTAGCTCAGTCCTAGGGATTATGCTAGCCATATCGAAAGGATAGTCTTGATAACCATAAGTTTAATTAAA | BBa_J23113 |
| 36 | AGCTTTTAATTAAACTTATGGTTATCAAGACTATCCTTTCGATATGGCTAGCATAATCCCTAGGACTGAGCTAGCCATCAGT | BBa_J23113 |
| 37 | ATGCAT**ACTAGT**CTGATGGCTAGCTCAGTCC | BBa_J23113 |
| 38 | TGATAACCATAAGTTTAATTAAATGACAAAGTATGCATTAGTCGGT | P_pc113_-*glk* |
| 39 | **GCGGCCGCTCTAGA**GTAGCGGTAAACGGCAGACAAAAAAAATGTCGCACAATGTGCGCCATTTTTCACTTCACAGGTTTACAGAATGTGACCTAAGGTCTG | *glk*-T_lpp_ |
| 40 | TGATAACCATAAGTTTAATTAAATGCCTGACGCTAAAAAACAGG | P_pc113_-*galP* |
| 41 | **GCGGCCGCTCTAGA**GTAGCGGTAAACGGCAGACAAAAAAAATGTCGCACAATGTGCGCCATTTTTCACTTCACAGGTTTAATCGTGAGCGCCTATTTCG | *galP*-T_lpp_ |
| 42 | ATGCAA**GCGGCCGCTCTAGA** | *Xba*I-*Not*I |
| 43 | ACCGGAACTATTGAGATCATTGCTCCGCTCTCTGGCGAGATCGTCAATAGACACTATAGAACGCGGCCG | *crr*-F1 |
| 44 | TGGGTTTGTTCGATAAACTGAAATCTCTGGTTTCCGACGACAAGAAGGATACCGGAACTATTGAGATCA | *crr*-F2 |
| 45 | CAGTTTGATCAGTTCTTTGATTTCGTCCATGTTGGAGATAACAACCGGACCGCATAGGCCACTAGTGGA | *crr*-R1 |
| 46 | TACTTCTTGATGCGGATAACCGGGGTTTCACCCACGGTTACGCTACCGGACAGTTTGATCAGTTCTTTG | *crr*-R2 |
| 47 | CCCAATGGCATCGTAAAGAACA | CM-RC |
| 48 | TGCTCAACCGACAACGGACG | *crr*-FC |
| 49 | AAGTGATGCGCTACACCCAG | *crr*-RC |
| 50 | CTCGATCTACTCGTGCTAAGAGGCATTGATTTACGCGGTATTGAGATTGGACACTATAGAACGCGGCCG | *tyrR*-F1 |
| 51 | TGCGTCTGGAAGTCTTTTGTGAAGACCGACTCGGTCTGACCCGCGAATTACTCGATCTACTCGTGCTAA | *tyrR*-F2 |
| 52 | ATCGCGGTATGTGAAACGCCGAGACGTTTTGCCAGTTTGCGCGTGCTGCCGCATAGGCCACTAGTGGA | *tyrR*-R1 |
| 53 | TTACTCTTCGTTCTTCTTCTGACTCAGACCATATTCCCGCAACTTATTGGCAATCGCGGTATGTGAAACG | *tyrR*-R2 |
| 54 | GCAAAACGCCCAGCGAAAAATAAT | *tyrR*-FC |
| 55 | AATTGCTGATGGCCCACTG | *tyrR*-RC |
| 56 | TCGCGACAATCCCACCGCGCTTTTTCACCAGTTGTGTGGGGATCGTCCGGGACACTATAGAACGCGGCCG | *trpE*-F1 |
| 57 | GCAAACACAAAAACCGACTCTCGAACTGCTAACCTGCGAAGGCGCTTATCGCGACAATCCCA | *trpE*-F2 |
| 58 | CGCGGGCTTTGTTACGGGTTTCGTCGGCTTCCGACTGCGGAACAGAATCACCGCATAGGCCACTAGTGGA | *trpE*-R1 |
| 59 | TCAGAAAGTCTCCTGTGCATGATGCGCGGTGGCAATAGCGCGCAGTACAGCGCGGGCTTTGTTACGG | *trpE*-R2 |
| 60 | TAGTACGCAAGTTCACGTAAAAAGGG | *trpE*-FC |
| 61 | ACAACCGGCTTCGCTCG | *trpE*-RC |
| 62 | AGCGCTGCTGAATTTATTAGCGAAGCGTCTGGAACTGGTTGCTGAAGTGGGACACTATAGAACGCGGCCG | *tyrA*-F1 |
| 63 | ATGGTTGCTGAATTGACCGCATTACGCGATCAAATTGATGAAGTCGATAAAGCGCTGCTGAATTTATTAG | *tyrA*-F2 |
| 64 | GAAAACGCTGTGCGTAATCGCCGAACCAGTGCTCCACCTTGCGGAAACTGCCGCATAGGCCACTAGTGGA | *tyrA*-R1 |
| 65 | TACTGGCGATTGTCATTCGCCTGACGCAATAACACGCGGCTTTCACTCTGAAAACGCTGTGCGTAATCG | *tyrA*-R2 |
| 66 | ATCCACGAGGGCAATCAGTC | *tyrA*-FC |
| 67 | GATTCACGGTAATCCATGGGAA | *tyrA*-RC |
| 68 | AACAGATCGCGATAATAATCTTGAAAAAGTTATCGCGGCGGGTGCCAACGGACACTATAGAACGCGGCCG | *pykA*-F1 |
| 69 | ATGTCCAGAAGGCTTCGCAGAACAAAAATCGTTACCACGTTAGGCCCAGCAACAGATCGCGATAATAATCGACACTATAGAACGCGGCCG | *pykA*-F2 |
| 70 | TCACGTCGCCCTGGGTGACAATCACCAGGTCACCAGACATCAAGTAACCTCCGCATAGGCCACTAGTGGA | *pykA*-R1 |
| 71 | TACTCTACCGTTAAAATACGCGTGGTATTAGTAGAACCCACGGTACTCATCACGTCGCCCTGGGTGACA | *pykA*-R2 |
| 72 | GAAGCGGGTCAAAGAAGCG | *pykA*-FC |
| 73 | GCTGAAAACTCGCAAACCGG | *pykA*-RC |

^a^ Restriction enzyme sites are shown in bold.
