# Supplementary material for: Metabolic engineering of Escherichia coli for the production of cinnamaldehyde
Source: Microb Cell Fact. 2016 Jan 19;15:16. doi: 10.1186/s12934-016-0415-9 (PMC4719340; doi:10.1186/s12934-016-0415-9)
Supplement: Supplementary file 9 — 10.1186/s12934-016-0415-9 Designed 5′-UTR sequences and predicted expression level. [file 12934_2016_415_MOESM9_ESM.docx]

**Additional file 9: Table S2. Designed 5*'*-UTR sequences and predicted expression level**

| Gene | 5*'*-UTR sequence | dG_UTR_ | Predicted level |
| --- | --- | --- | --- |
| *aroG*8/15 | CGACAAAAAGAAAGGAGCATCTAAC | -9.64 | 1745888.41 |
| *ydiB* | AAAACCCCACAAAGGAGCATCAAAG | -4.79 | 115863.62 |
| *aroK* | AAAAAACAGAAAAGGAGCATCGTGG | -7.99 | 693793.12 |
| *pheA^fbr,dm^* | AAAAAAGAAAAAAGGAGCATCTTTG | -6.69 | 335319.50 |
