# Supplementary material for: Metabolic engineering of Escherichia coli for the production of cinnamaldehyde
Source: Microb Cell Fact. 2016 Jan 19;15:16. doi: 10.1186/s12934-016-0415-9 (PMC4719340; doi:10.1186/s12934-016-0415-9)
Supplement: Supplementary file 10 — 10.1186/s12934-016-0415-9 Strain construction. [file 12934_2016_415_MOESM10_ESM.docx]

**Additional file 10: Strain construction**

1. Chromosomal gene deletion was performed using rapid one-step inactivation method. The integrated knockout system vector (pCW611) was used to disrupt the desired genes in the chromosomal DNA of *E. coli* W3110 with Cm^R^ marker (chloramphenicol-resistance) for selection. The recombinant *E.coli* W3110 harboring the pCW611 was cultivated at 30^o^C with adding 0.2% (w/v) arabinose (13.3 mM) for induction of λ-red recombinase system. And then, electro-competent cells were prepared with standard protocol. For knockout experiment of each genes, the linear gene knockout DNA fragments were amplified by using pEcmuloxC plasmid which contains the *loxLE*-Cm^R^ gene-*loxRE* region as a template. And homologous arm regions of each genes were extended by two-step PCR using the primer combinations F1/R1, F2/R2 of each gene. Approximately 3 μg of linear gene knockout DNA fragment was transformed into the 100 μL of electro-competent cells harboring the pCW611 plasmid by electroporation method. After that, recombination event occurred colonies were incubated at 30^o^C and selected on LB agar plate containing 17.5 μg/mL of Cm. The recombinants with Cm^R^ gene were confirmed by colony PCR with CM-RC primer and FC primer of each genes. The confirmed recombinant colony was directly streaked on the LB agar plate containing 17.5 μg/mL Cm and 100 μg/mL ampicillin (Amp). And the Cm^R^ antibiotic marker was easily removed by spreading on the LB agar plate containing 100 μg/mL Amp and 1 mM IPTG (isopropyl-β-D-thiogalactopyranoside) to excise the Cm^R^ gene. Subsequent to excision step of Cm^R^ gene, we finally checked the complete deletion of desired gene by using the primer combination FC/RC of each genes. Above procedures had been iterated until making the recombinant *E. coli* YHP05 strain. When all gene deletions are finished, pCW611 vector was easily cured by growing at 37^o^C or 42^o^C because of a temperature-sensitive origin of replication. Before we cultivated the knock out mutants, all knock out genotype were checked again by PCR with FC/RC primers of each genes.
